# Along the *Bos taurus* genome, uncover candidate imprinting control regions

**DOI:** 10.1186/s12864-022-08694-3

**Published:** 2022-06-28

**Authors:** Phillip Wyss, Carol Song, Minou Bina

**Affiliations:** 1 Department of Chemistry, Purdue University, West Lafayette, IN 47907 USA; 2 Information Technology, Purdue University, West Lafayette, IN 47907 USA

**Keywords:** Cattle genomics, CNR1, DGAT1, HMGA2, KAP1, KMT2A, KMT2B, LCORL, NR4A1, PTEN, ZFP57

## Abstract

**Background:**

In mammals, Imprinting Control Regions (ICRs) regulate a subset of genes in a parent-of-origin-specific manner. In both human and mouse, previous studies identified a set of CpG-rich motifs occurring as clusters in ICRs and germline Differentially Methylated Regions (gDMRs). These motifs consist of the ZFP57 binding site (ZFBS) overlapping a subset of MLL binding units known as MLL morphemes. MLL or MLL1 (Mixed Lineage Leukemia 1) is a relatively large multidomain protein that plays a central role in the regulation of transcription. The structures of both MLL1 and MLL2 include a domain (MT) that binds CpG-rich DNA and a conserved domain (SET) that methylates lysine 4 in histone H3 producing H3K4me3 marks in chromatin.

**Results:**

Since genomic imprinting impacts many developmental and key physiological processes, we followed a previous bioinformatics strategy to pinpoint ICR positions in the *Bos taurus* genome. Initial genome-wide analyses involved finding the positions of ZFP57 binding sites, and the CpG-rich motifs (ZFBS-morph overlaps) along cattle chromosomal DNA. By creating plots displaying the density of ZFBS-morph overlaps, we removed background noise and thus improved signal detection. With the density-plots, we could view the positions of peaks locating known and candidate ICRs in cattle DNA. Our evaluations revealed the correspondence of peaks in plots to reported known and inferred ICRs/DMRs in cattle. Beside peaks pinpointing such ICRs, the density-plots also revealed additional peaks. Since evaluations validated the robustness of our approach, we inferred that the additional peaks may correspond to candidate ICRs for imprinted gene expression.

**Conclusion:**

Our bioinformatics strategy offers the first genome-wide approach for systematically localizing candidate ICRs. Furthermore, we have tailored our datasets for upload onto the UCSC genome browser so that researchers could find known and candidate ICRs with respect to a wide variety of annotations at all scales: from the positions of Single Nucleotide Polymorphisms (SNPs), to positions of genes, transcripts, and repeated DNA elements. Furthermore, the UCSC genome browser offers tools to produce enlarged views: to uncover the genes in the vicinity of candidate ICRs and thus discover potential imprinted genes for experimental validations.

**Supplementary Information:**

The online version contains supplementary material available at 10.1186/s12864-022-08694-3.

## Background

For centuries, breeders have relied on principles of inheritance to obtain animals with desired traits [1–7]. However, emerging data indicate that assisted reproductive technologies (ART) may induce fetal overgrowth producing LOS –large offspring syndrome [8–15]. Partly, developmental anomalies arose from altered DNA methylation patterns causing imprinting defects in ICRs, inferred DMRs, or both [1, 2, 11, 12, 14]. Furthermore, researchers observed that Somatic Cell Nuclear Transfer (SCNT) procedures, could epigenetically disturb imprinted gene expression [11].

Overall, genome imprinting is relatively complex and requires orchestrated action of several proteins, including ZFP57, KAP1/TRIM28, and a subset of DNA methyltransferases [16–20]. In ICRs/gDMRs, ZFP57 recognizes its methylated hexameric site [21] and thus plays a central role in the establishment of genomic imprints [16, 21, 22]. ZFP57 family members (KZFPs) are encoded in the hundreds by the genomes of higher vertebrates [23, 24]. Most KZFPs are essential to the recruitment of KAP1 and associated effectors to chromatin to repress transcription [23]. ZFP57 is necessary to maintaining the DNA methylation memory at multiple ICRs in mice embryos and embryonic stem cells [16, 21, 22, 25]. In addition to ZFP57 binding sites, the ICRs often include closely-spaced ZFBS-morph overlaps [26]. These overlaps consist of ZFP57 binding site overlapping a subset of the MLL1 binding units known as morphemes [27]. Since these units are CpG-rich, they are spread across the CpG islands. In the islands that encompass coding exons, the morphemes impact codon utilization [28]. Overall, in the human genome, CpG-rich promoters encompass many CpG-rich motifs with potential regulatory characteristics [29, 30].

ZFBS-morph overlaps are composite-DNA-elements that could play dual but antagonistic roles in the regulation of allele-specific gene expression: ZFP57 binding to its methylated sites to maintain allele-specific gene repression; binding of MLL1 or MLL2 to CpG-rich sequences to protect ICRs from methylation to support transcription [26]. MLL1 is the founding member of a protein family whose structure includes a conserved domain (SET) that catalyzes methylation of H3K4 (lysine 4 in histone H3) producing H3K4me3 marks in nucleosomes [31]. Trimethylated H3K4me3 is associated with active or transcriptionally poised chromatin states [32]. In addition to the SET domain, the structures of MLL1/KMT2A and MLL2/KMT2B include a domain (MT or CXXC) that binds unmodified CpG-rich DNA [33–35]. De novo mutations in *MLL1* cause Wiedemann-Steiner syndrome [36]. Symptoms vary and may include delayed growth and development, asymmetry of the face, hypotonia, and intellectual disability [36]. Mutations in *MLL2* cause complex early-onset dystonia [37, 38]. In mouse oocytes, MLL2 was required for bulk H3K4 trimethylation [39].

In both mouse and human DNA, known ICRs/gDMRs encompass clusters of two or more ZFBS-morph overlaps [40, 41]. Therefore, we wished to investigate whether known or inferred cattle ICRs/DMRs also included these composite-DNA-elements for regulating parent-of-origin-specific expression. To do so, we performed genome-wide analyses of *Bos taurus* chromosomal DNA sequences. Firstly, we located ZFP57 binding sites and ZFBS-morph overlaps. Subsequently, we created density-plots to pinpoint ICR positions in *Bos taurus* DNA. By uploading our datasets onto the UCSC genome browser, we could obtain snapshots to view peak positions with respect to genomic landmarks. These snapshots uncovered a connection between peaks in plots and the ICRs in cattle imprinting domains including *H19—IGF2*, *KCNQ1*, *IGF2R*, and *PEG3*. Additional snapshots revealed such a connection for: the essential ICR in the *GNAS* complex locus; and ICRs in *PLAGL1*, *MEST*, *NNAT*, *MEG8*, *SNRPN*, *HERC3-NAP1L5*, and *INPP5F*, *loci*. Since peaks in plots could locate known ICRs/DMRs in *Bos taurus*, we anticipate that with our approach one could discover candidate ICRs and novel imprinted genes for experimental validations.

## Results

Our datasets consist of genomic positions of ZFP57 binding site, ZFBS-morph overlaps, and peaks in the density-plots. To evaluate the power of our strategy, we have closely inspected peak positions in genomic sequences reported for mouse, human, and *Bos taurus* (Table 1). In addition to previously published reports [26, 40, 41], we have also checked peak positions with respect to experimental data reported in GEO series GSE77444. Briefly, the experimental approach was to use mouse embryonic stem cells (ESCs) E14 to locate regions associated with ZFP57, KAP1, and H3K9me3 marks in chromatin [25]. Such associations would reveal the positions of ICRs/gDMRs in genomic DNA [22, 25]. Noteworthy could be that for the build mm9 of the mouse genome, GSE77444 also included a link for viewing the individual data series in tracts displayed on the UCSC genome browser [25]. Additional file 1 gives snapshots demonstrating that peaks in the density-plots occur precisely in regions associated with ZFP57, KAP1, and H3K9me3 marks in chromatin prepared from mouse ESCs. Based on these and other evaluations, we imagined that with our strategy, one could locate known and candidate ICRs in mammalian DNA. To further investigate this idea, initially we give examples of the positions of ZFP57 binding site, ZFBS-morph overlaps, and peaks in the density-plots in the context of known ICRs or inferred DMRs dispersed along the *Bos taurus* genome. Afterward, we cover examples illustrating that with our strategy we could discover novel candidate ICRs and imprinted genes in cattle DNA.

**Table 1:** Peaks in the density-plots locating known or inferred ICRs in 3 mammalian species

Genomic *loci*	ICRs/gDMRs Mouse	ICRs human	DMRs *Bos taurus*
*Gpr1-Zdbf2*	Y	N	A
*Gnas*	Y	Y	Y
*Mcts2*	Y	N	N
*Nnat*	Y	Y	Y
*Mest iso*	Y	Y	Y
*Nap1l5*	Y	Y	Y
*Sgce-Peg10*	Y	N	Y
*Rasgrf1*	Y	N	N
*H19-Igf2*	Y	Y	Y
*Kcnq1 locus*	Y	Y	Y
*Inpp5f_v2*	Y	Y	Y
*Peg3*	Y	Y	Y
*Snrpn*	Y	N	Y
*Plagl1 iso./Zac1*	Y	Y	Y
*Grb10*	Y	Y	A
*Zrsr1*	N	N	C
*IG-DMR*	Y	N	A
*Peg13*	Y	N	C
*Igf2r-Airn*	Y	N	Y
*Impact*	N	N	A

*Y* yes, *N *no, *A *ambiguous, *iso *isoform, *C* comment

In the build BosTau8, the browser did not locate Peg13 and Zrsr1

The build of analyzed genomes consisted of: mouse, mm9; human, hg19; Bos taurus, BosTau8

### Along Bos taurus chromosomes, peaks in the density-plots pinpointed known, unknow, and inferred ICRs/DMRs

In mice, the expression of *H19*, *Igf2*, and *Ins2* is regulated by a single ICR/gDMR positioned upstream of *H19* [18]. The noncoding RNA gene (*H19*) is transcribed from the maternal allele. *Igf2*, and *Ins2* are expressed from the paternal allele and impact fetal growth and body size. As in mice, the *H19*—*IGF2* imprinted domain is important to normal growth and fetal development in cattle [2, 11, 42, 43]. In cloning studies, deceased newborn calves displayed abnormal expression of *H19* and *IGF2*. In normal surviving adults, the expression of *IGF2* in muscle was highly variable [44]. Furthermore, aberrant methylation of a DMR upstream of *H19* produced abnormal calves and LOS [11, 45]. In *H19* DMR, a study found 33 CpGs in 600 bps [11]. Another study detected a 300-bp DMR at approximately 6 kb upstream of the *H19* promotor [46]. This DMR mapped to a predicted CpG island with a CTCF-binding site corresponding to consensus sequence 5’ -GCGGCCGCGAGGCGGCAGTG- 3’ [46].

**Fig. 1:**
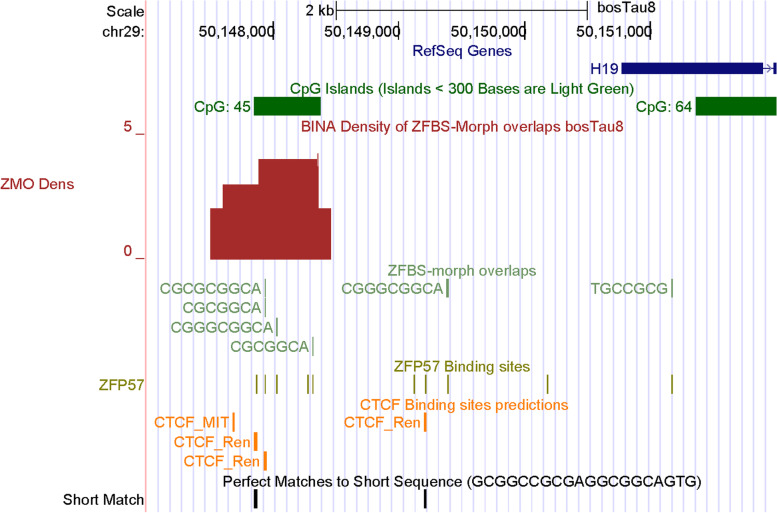
A robust peak identifying the ICR of the *H19* — *IGF2* imprinted domain. From top to bottom, tracks display the positions of: cattle RefSeq genes, in pack format; the CpG islands, in pack format; peaks in density-plot, in full format; ZFBS-morph overlaps, in pack format; ZFP57 binding sites, in dense format. Orange bars mark the positions of predicted CTCF binding sites. Short match corresponds to a previously reported consensus CTCF binding sequence

In the density-plot of Chr29, we noticed a robust peak in a CpG island (CpG45) upstream of *H19* (Fig. 1). Peak position agrees with the reported DMR in cattle DNA [46]. Furthermore, the length of CpG45 is about the same as the CpG-rich DNA (600 bps) selectively methylated in cattle paternal allele [11]. Additionally, under the density-peak, we located 3 predicted CTCF sites (Fig. 1). In the analyzed DNA segment, one site (CTCF_Ren) corresponds to the consensus sequence (GCGGCCGCGAGGCGGCAGTG) identified previously [46]. Three sites are under the density peak and map to the CpG island that includes the experimentally identified cattle DMR [46]. The 5th site is downstream of CpG45 (Fig. 1). Thus, we inferred that our approach correctly located the ICR in cattle *H19 —IGF2* imprinted domain.

As in mice [47], the *KCNQ1* imprinted domain is adjacent to the *H19*—*IGF2* domain and regulated by an ICR Known as the KvDMR1 in cattle [48]. In mice, the KvDMR1 encompasses the *Kcnq1ot1* promotor and regulates imprinted expression of several protein-coding genes [42, 47, 49, 50]. This intragenic ICR is selectively methylated in oocytes but not in sperm [42, 51]. Thus, while *Kcnq1ot1* is transcribed from the paternal allele producing a noncoding RNA, the expression of several protein coding genes is repressed in the maternal allele [42]. In mice, targeted deletion of the KvDMR1 caused loss of imprinting and growth deficiency [52]. In cattle, defects in the KvDMR are the most common genomic region affected in LOS [8, 12, 15, 45, 48].

**Fig. 2:**
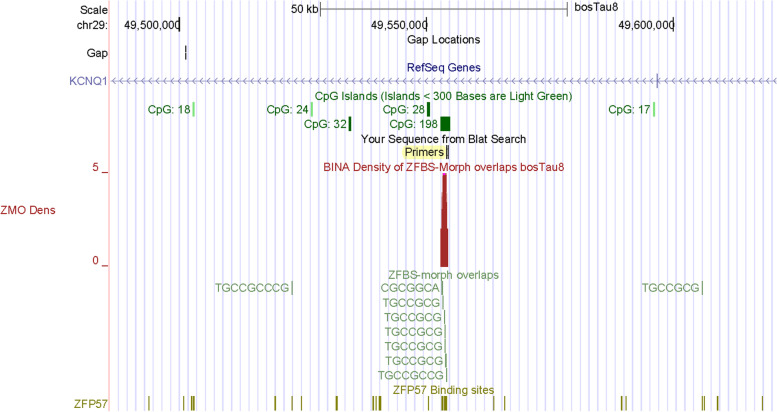
A robust peak pinpointing the KvDMR1. Previously, a DNA segment was amplified for locating the KvDMR1 in bovids. The plot shows the position of the two primers selected for amplification reaction. ZFBS-morph overlaps are shown in packed and ZFP57 binding sites in dense formats

In the density-plots of *Bos taurus* DNA, we observed a very robust peak in one of the *KCNQ1* introns (Fig. 2). In order to ensure that this peak was locating the KvDMR1, initially we selected the 5’ and 3’ ends of human *KCNQ1OT1* to perform a BLAT search at the UCSC genome browser. Due to length constraints imposed by BLAT, we could not use the entire human *KCNQ1OT1* as query. Nonetheless, from the BLAT output we could deduce that the peak was in the vicinity of *KCNQ1OT1*. For additional validation, we examined results of a study that localized cattle KvDMR1 using 2 primers to amplify an intragenic DNA. In that study, statistical analysis showed a significant difference in the methylation level between the two parental alleles –confirming that the amplified DNA corresponded to the KvDMR1 [53]. Therefore, we performed another BLAT search using the sequences of the 2 primers selected for amplifying an intragenic DNA [53]. A close-up view shows that the primers are within a relatively long CpG island that encompasses the robust peak in the density-plots (Fig. 2). Thus, that peak located the central position of the KvDMR in cattle. Notably, while the KvDMR1 in mice encompasses 2 ZFBS-morph overlaps [26], that in cow encompasses 7. Therefore, it seems that ICRs display species-specific differences in the number of ZFBS-morph overlaps they encompass.

**Fig. 3:**
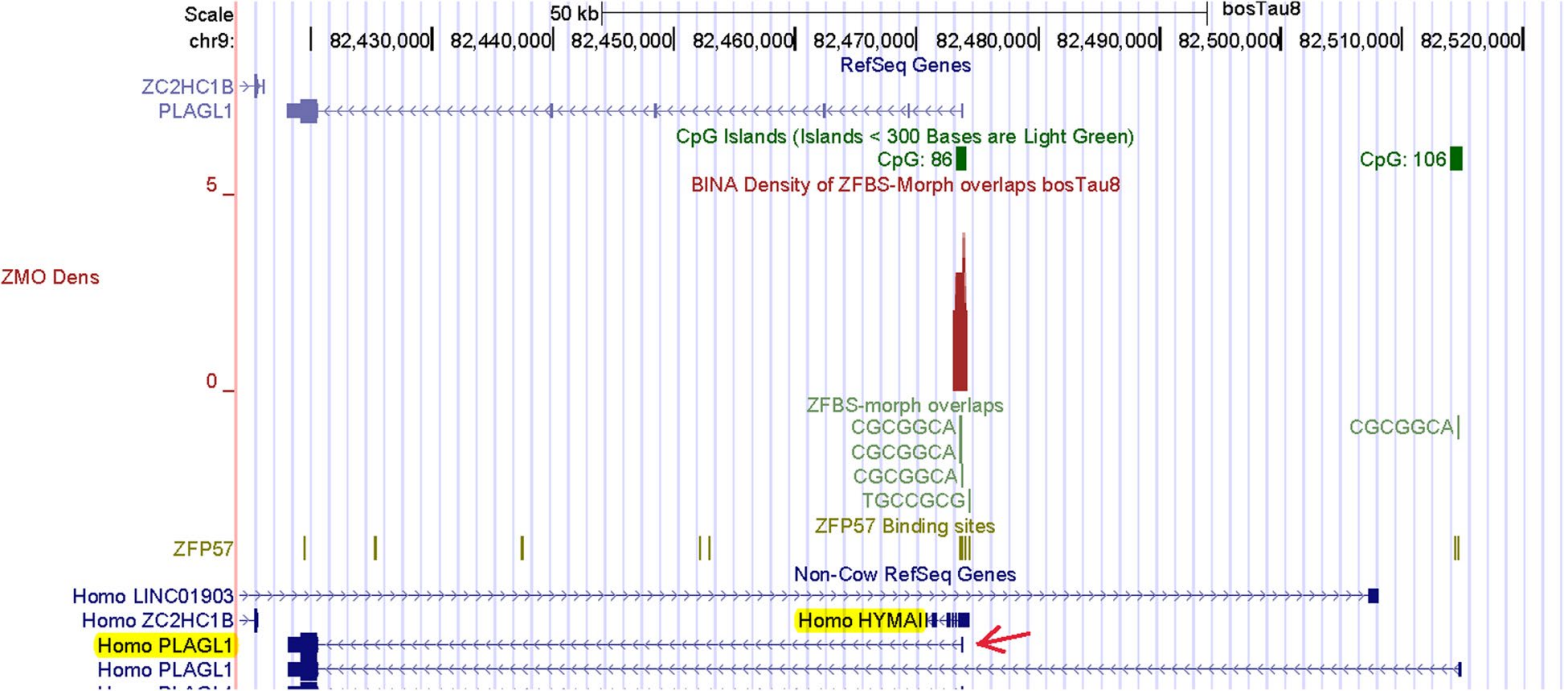
A robust peak locating the ICR for imprinted expression of the *PLAGL1* transcript known as *ZAC1*. The peak is at the correct position with respect to two human imprinted transcripts: one corresponding to *HYMAI*; the other to *ZAC1*

Next, we examined the density-plots in a DNA segment that encompasses the *PLAGL1 locus* (Fig. 3). Comparative analyses have identified a group of transcription factors with a zinc finger at their amino-terminus [54]. This group includes PLAG1, PLAGL1, and PLAGL2. One of the *PLAGL1* transcripts (*ZAC1*) encodes a protein that inhibits tumor-cell-proliferation through the induction of cell cycle arrest and apoptosis [55]. In contrast, *PLAG1* and *PLAGL2* are proto-oncogenes [55]. *ZAC1* is an intragenic maternally imprinted transcript [56–58]. In both the mouse and human genomes, transcription of *ZAC1* originates within an intronic sequence in *PLAGL1*. The intronic DNA includes another imprinted gene known as *HYMAI* [26, 57, 58]. Both *ZAC1* and *HYMAI* are selectively expressed from the paternal allele. Their ICR/gDMR corresponds to an intragenic CpG island [26, 57, 58]. When the gDMR from human DNA was transferred into mice, it acted as an ICR and regulated allele-specific expression [59]. In LOS induced by assisted reproduction, imprinting was compromised producing biallelic expression [8, 13].

As previously observed in mice [60], *PLAGL1* locus in cattle encompasses a cluster of ZFBS-morph overlaps. In the density-plots, that cluster defines a peak (Fig. 3). However, instead of several *PLAGL1* transcripts, cattle RefSeq Genes displayed only one. We suspected that this transcript corresponded to a previously reported annotation. Specifically, in a panel of bovine imprinted genes, *PLAGL1* was among the *loci* that acquired methylation marks in an oocyte size-specific manner [61]. In that panel, a putative DMR was localized in a CpG island at the 5’ end of a single transcript referred to as *PLAGL1*/*ZAC1* [61]. To obtain clues about a complete annotation, we inspected our predicted ICR-position with respect to Non-Cow RefSeq Genes. In the context of human RefSeq Genes, we noticed 2 *PLAGL1* transcripts and a noncoding RNA gene marked as *HYMAI* (Fig. 3). Therefore, it seems likely that *ZAC1* corresponds to the transcript annotated as *PLAGL1* in cattle RefSeq Genes. In that context, the peak in cattle DNA is intragenic –as observed in human genomic DNA [41]. Hence, we are tempted to conclude that our strategy has pinpointed the reported putative *ZAC1* DMR [61], and to deduce that in cattle *PLAGL1 locus*, the putative *ZAC1* DMR is a *bona fide* ICR (Fig. 3).

Next, we examined the density-plots in a region encompassing the *IGF2R*—*AIRN* imprinted domain. In mouse, the second intron of *Igf2r* lies the *Airn* promoter [62]. Also known as *Air*, *Airn* specifies an imprinted *cis*-silencing noncoding transcript [62]. *Igf2r* (insulin-like growth-factor type-2 receptor) is maternally expressed and impacts fetal and placental growth [63, 64]. Its functions include transport of IGF2 into cells and to lysosomes for degradation [65]. In mice, *Igf2r* knockout caused fetal overgrowth and neonatal lethality [66]. The *Igf2r—Airn* imprinted domain includes two differentially methylated CpG islands [64]. DMR1 encompasses the *Igf2r* promotor. The CpG island that encompasses DMR2 is intragenic. In this island is incorporated the promotor of *Airn* [67]. DMR2 regulates expression of *Igf2r* from the maternal and *Airn* from the paternal allele [68]. In mice, deletion of DMR2 caused biallelic *Igf2r* expression [67]. In cattle, The *IGF2R—AIRN* imprinted domain also includes the DMR2 that regulates imprinted expression. In SCNT experiments, *IGF2R* expression was consistently biallelic –regardless of the source of cattle embryos [11].

**Fig. 4:**
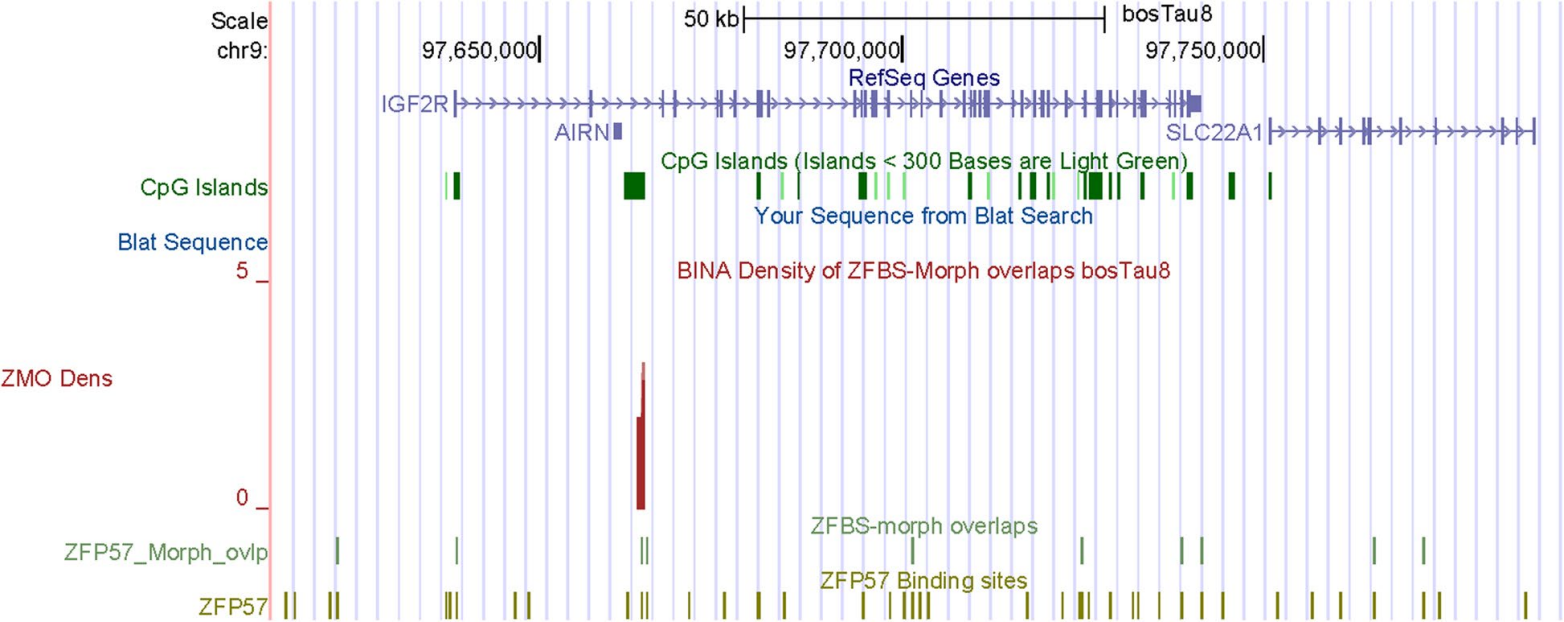
A robust peak defining the ICR of *IGF2R* imprinted domain in cattle genome. Displayed in packed formats are the genomic positions of the CpG islands, ZFBS-morph overlaps, and ZFP57 binding sites

While the *IGF2R—AIRN* imprinted domain in *Bos taurus* encompasses many CpG islands, one could assume that DMR1 corresponds to the island at the 5’ end of *IGF2R* and DMR2 to the intragenic island that maps to the *AIRN* promotor (Fig. 4). In that context, in the density-plots clearly apparent is a robust peak in the CpG island at the *AIRN* promotor (Fig. 4). Since this peak is in DMR2, we deduce that our approach has pinpointed the ICR in the *IGF2R—AIRN* imprinted domain in cattle. Notably, while the gDMR in the mouse genome encompasses 7 ZFBS-morph overlaps [26], the gDMR in *Bos taurus* encompasses 3. Thus, as mentioned above, the number of ZFBS-morph overlaps in ICRs could be species-specific.

**Fig. 5:**
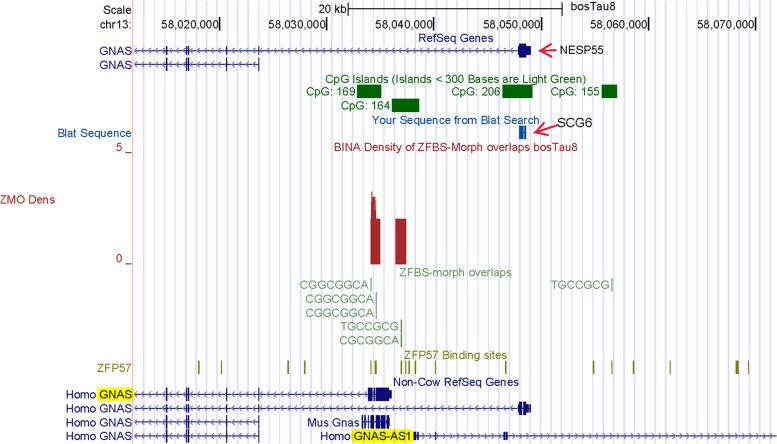
Peaks predicting the position of the essential ICR in the *GNAS* complex locus. The arrow points to result of a BLAT search identifying the longer *GNAS* transcript as *NESP55* (encoding SCG6). The predicted ICR position seems correct with respect to the annotation of Non-Cow RefSeq Genes including human *GNAS-AS1*

Next, we examined the density-plots in a region encompassing the complex *GNAS locus* (Fig. 5). In both the human and mouse genome, the *GNAS locus* encompasses multiple DMRs, promotors, and allele-specific transcripts [69]. Since several transcriptional variants are produced from differential exon utilization, they are collectively referred to as *GNAS*. The well-studied *Gnas locus* in mice includes three groups of protein-coding transcripts (*Nesp55*, XL*as*, and G*as*). Although these transcripts share alternative exons, they are regulated by separate promotors [69, 70]. Among the transcripts: *Nesp55* is expressed from the maternal allele; XL*as* from the paternal allele; G*as* from both alleles [70]. *Nesp55* specifies a neuroendocrine secretory protein known as SCG6. XL*as* and G*as* transcripts are related. Their products function in signal transmission, by G coupled hormone-receptors (GPCRs), and display distinguishable properties [71–74]. The *locus* also includes an imprinted gene (*Nespas*) transcribed into a noncoding antisense RNA [69].

At the genome browser, we noticed that in contrast to complete annotations reported for mouse and human genomic DNA, cattle RefSeq genes displayed only two transcripts (Fig. 5). From a publication [75], we inferred that the shorter transcript was likely to encode the G*a*s subunit of GPCRs. In cattle, SNPs within that transcript were associated with performance traits [75]. Even though not displayed on the browser, for cattle a previous study identified *GNASXL* as a paternally expressed transcriptional isoform. The study also described a maternally expressed transcript designated as *GNAS* or *NESP55* [76].

Because the UCSC genome browser’s gene annotations appeared incomplete, we performed a BLAT search to locate *NESP55* in *Bos taurus* DNA. For query we chose the amino acid sequence of human SCG6. Based on the output, we inferred that on the browser, the longer *GNAS* transcript corresponded to *NESP55* (Fig. 5). Cattle *NESP55* transcripts are expressed monoallelically in many tissues [76]. Furthermore, in cattle, the *GNAS* locus includes a DMR (a putative ICR) that is hypomethylated in the paternal allele and hypermethylated in the maternal allele [76].

In the density-plots of *GNAS* locus in cattle, we observed two clusters of ZFBS-morph overlaps producing two peaks in *Bos taurus* DNA (Fig. 5). Similarly, a previous study also noticed two clusters of ZFBS-morph overlaps in the mouse locus [26]; also see Additional file 1. In cattle, both peaks are in the 1^st^ intron of the transcript corresponding to *NESP55* (Fig. 5). Notably, the essential ICR in human locus includes two DMRs: one DMR maps to the first exon of *XLas*; the other is near *GNAS-AS1* TSS [69]. With respect to human RefSeq Genes, peak positions in cattle DNA map to human *XLas* and to a region upstream *GNAS-AS1* (Fig. 5). *In toto*, we could infer that our strategy predicted the genomic position of the essential ICR in the complex *GNAS locus* in *Bos taurus*.

**Fig. 6:**
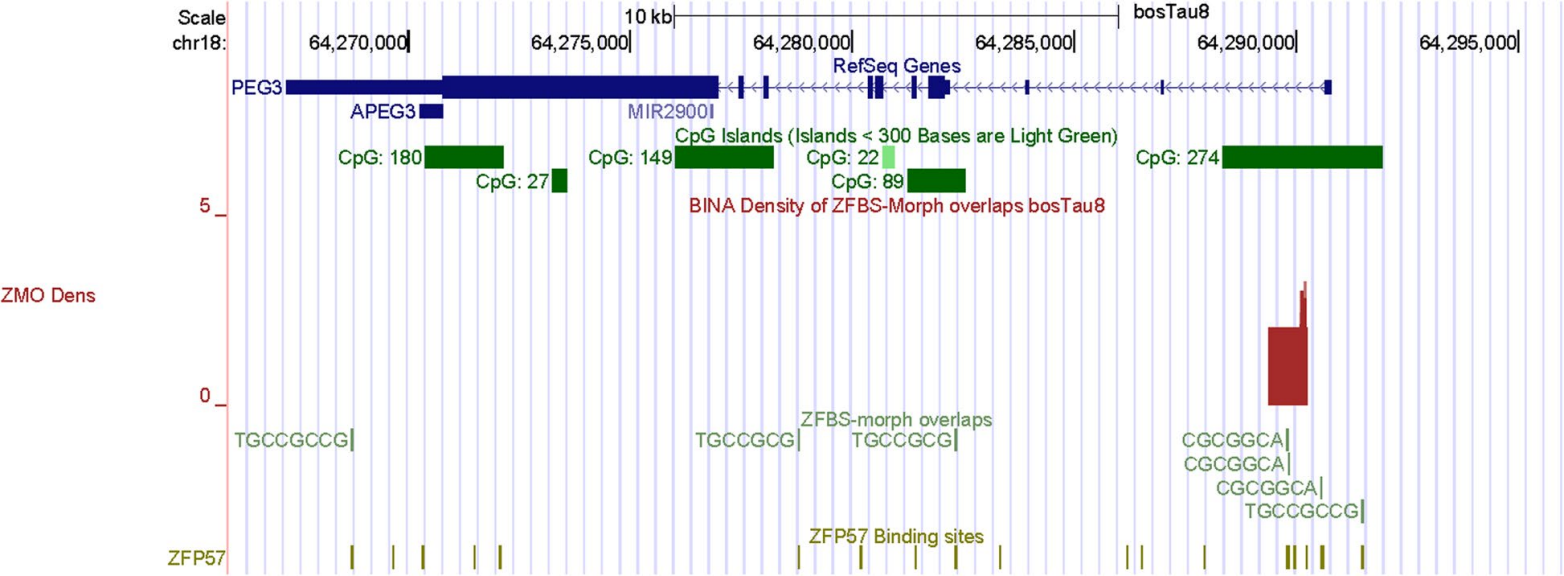
A robust peak locating the ICR in the *PEG3* imprinted domain. As detailed in the text, this peak is within the CpG island that is methylated in a parent-of-origin-specific manner

Next, we inspected the *PEG3* imprinted domain in *Bos taurus* DNA (Fig. 6). Sequence analyses have predicted that the *PEG3* domain encompasses many CpG islands [77]. Consistent with this prediction, at the genome browser we observed several CpG islands (Fig. 6). The island encompassing the *PEG3* promotor was the only area that showed DMR status in cattle [77]. In that area, the density-plots revealed a peak (Fig. 6). Since the DMR was methylated in an allele-specific manner [77], we deduced that the peak pinpointed the ICR in cattle *PEG3* imprinted domain. Furthermore, earlier studies of mice, revealed that clusters of ZFBS-morph overlaps mapped to functionally important landmarks –including DNase I hypersensitive sites and repressive H3K9me3 marks in chromatin [26]. In mouse, these clusters were in the 1^st^ *Peg3* intron. Likewise, for cattle we observe an intragenic peak. Therefore, our strategy pinpointed the central portion of the ICR in cattle *PEG3* imprinted domain (Fig. 6).

In mice, the *Peg3* domain is comprised of several genes [78]. The product of *Peg3* is a relatively large nuclear protein with 12 zinc fingers of C2H2 type [79]. In mice, a mutation in *Peg3* caused a striking impairment of maternal behavior [80]. Due to the dearth of maternal care, the litters developed poorly and often died [80]. Mechanistically, mutant mothers were deficient in milk ejection –partly due to defective neuronal connectivity, as well as reduced oxytocin neurons in the hypothalamus [80]. In domesticated animals, oxytocin is an indicator of psychological and social well-being [81]. As observed in mice and humans, *PEG3* is expressed from the paternal allele in cattle [2, 77]. As the consequence to assisted reproduction, the *PEG3* domain has undergone a global loss of imprinting producing LOS [8].

**Fig. 7:**
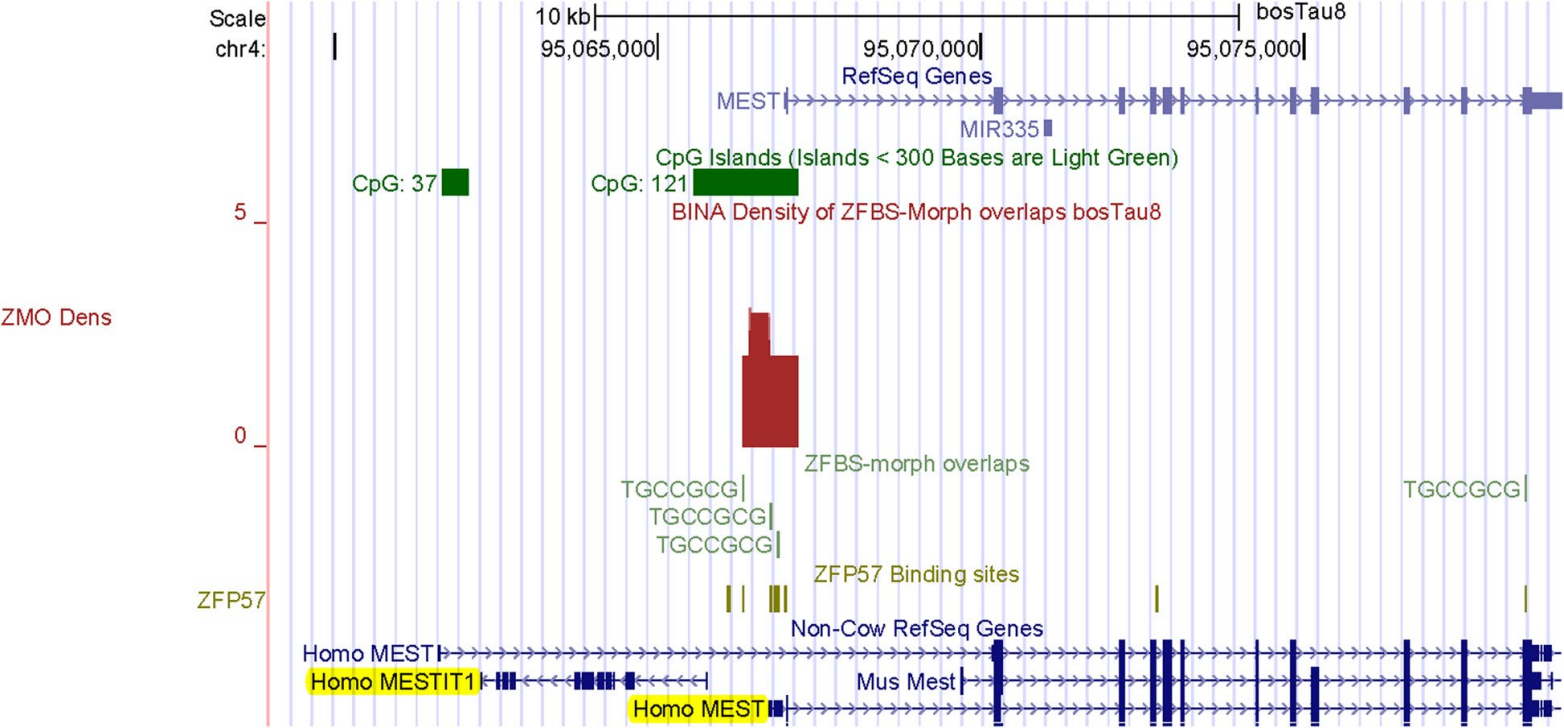
A robust peak locating the ICR for imprinted expression of the *MEST* variant. The peak position is correct in the context of Non-Cow RefSeq Genes for human *MESTIT1* and *MEST* transcriptional variant

Next, we examined the density-plots in a region encompassing the *MEST locus* (Fig. 7). In cattle, among 8 investigated genes, only *MEST* showed differential expression in day 21 parthenogenetic embryos [82]. In mice, the TSS of a paternally expressed transcript originates from the 1^st^ *Mest* intron [83, 84]. This transcript is also known as *Peg1* and regulated by an intragenic ICR.

In a panel of maternally imprinted genes in cattle, researchers localized putative DMRs in several imprinted *loci* including *MEST* [61]. By CpG analyses, they inferred that the putative *Peg1*/*MEST* DMR mapped to a CpG island [61]. Consistent with this prediction, the density-plots revealed a peak in a CpG island in the *MEST locus* (Fig. 7). Since on the browser we observed a single *MEST* transcript, we inspected peak position with respect to Non-Cow RefSeq Genes. In the context of human RefSeq Genes, the peak in plots is upstream of *MESTIT1* and near one of the *MEST* short isoforms. Also known as *PEG1-AS*, human *MESTIT1* is a paternally expressed non-coding RNA gene [85, 86]. Note that with respect to human MEST and *MESTIT1*, peak location agrees with the position of reported imprinted DMR in cattle [61]. Hence, we deduced that our strategy correctly pinpointed the ICR regulating the expression of *PEG1*/*MEST* transcript in *Bos taurus* (Fig. 7).

**Fig. 8:**
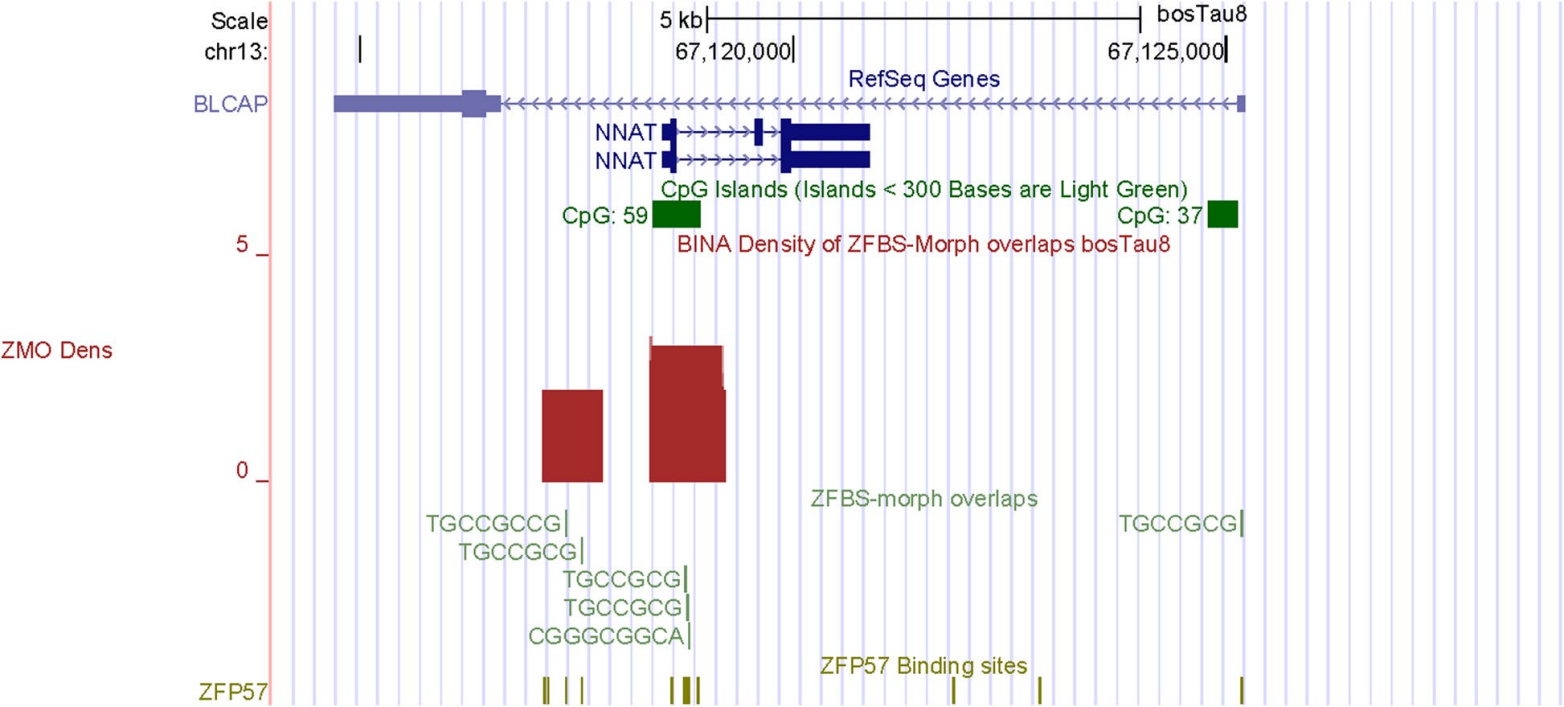
A robust peak locating a candidate ICR for imprinted *NNAT* expression. This peak is in CpG59 and maps to a known cattle DMR

Next, we examined the density-plots in a DNA segment that includes *NNAT* (Fig. 8). As in the mouse *locus* [7, 84], in cattle *NNAT* lies in the single *BLCAP* intron (Fig. 8). In mice, *Nnat* or *Peg5* is an imprinted gene; *Blcap* is expressed biallelically [7]. From *Nnat* are produced several alternatively spliced isoforms As in humans and mice [7, 87, 88], in cattle two *NNAT* transcripts are expressed from the paternal allele [89].

In cattle DNA: a CpG island (CpG37) is near the *BLCAP* TSS; another island (CpG59) is intragenic (Fig. 8) [7]. In the vicinity of *NNAT*, the density-plots includes two peaks. The robust one is in CpG59 and encompasses the *NNAT* promotor. In human, the CpG island at the *NNAT* promotor was differentially methylated in all examined tissues [88]. Therefore, CpG59 is likely position of the ICR that regulates imprinted *NNAT* expression in cattle DNA (Fig. 8). Overall, robustness of peaks depends on the number of ZFBS-morph overlaps they encompass. Peaks that cover 2 ZFBS-morph overlaps could be true or false positive [40]. Peaks that cover 3 are more reliable (Fig. 8).

**Fig. 9:**
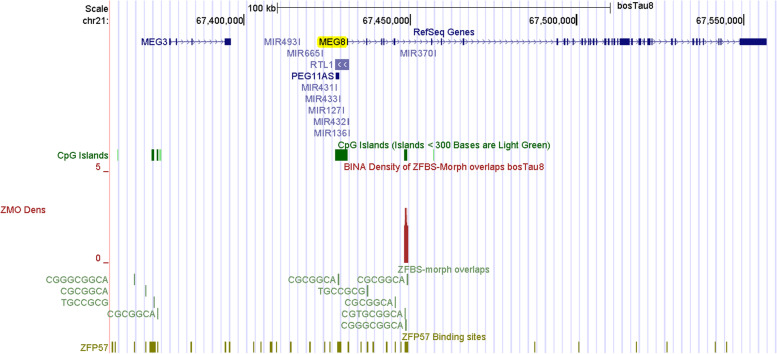
A robust peak predicting the ICR for imprinted expression of *MEG8*. This peak is intronic and maps to a CpG island

Next, we examined the density-plots in a region that encompasses *MEG8* (Fig. 9). In ovine, *MEG8* is expressed from the maternal allele producing a noncoding RNA [90]. In an 8-wk-old animal, *MEG8* was preferentially expressed in skeletal muscle [90]. In Angus calves, maternal diet during pregnancy impacted *MEG8* expression in longissimus dorsi muscle [91]. In adult cattle, *MEG8* was expressed in several tissues –including heart, liver, spleen, lung, kidney, brain, subcutaneous fat and skeletal muscle [92]. In heterozygous cattle, *MEG8* was expressed from only one of the two parental alleles suggesting that *MEG8* is an imprinted transcript [92]. In the density-plots, we observed a robust peak predicting a candidate ICR for allele-specific expression of *MEG8* in cattle. This peak is intragenic and maps to a CpG island (Fig. 9).

Furthermore, a peak in the density-plots located a candidate ICR for imprinted expression of *SNRPN* (Fig. S1). This peak in a CpG island that includes the *SNRPN* promoter, TSS, and first exon (Fig. S1). In cattle, *SNRPN* alleles were unmethylated in sperm, methylated in oocytes, and approximately 50% methylated in somatic samples [93]. When compared to in vivo and in vitro-produced embryos, the CpG island at the 5’ end of *SNRPN* was abnormally hypomethylated in SCNT-derived Day 17 elongating embryos [93].

A peak in the density-plots also located a candidate ICR for imprinted expression of *NAP1L5* in *Bos taurus* (Fig. S2). As in mouse [94], *NAP1L5* is paternally expressed in cattle [89]. In mouse, *Nap1l5* is entirely contained in the intron of another gene (*Herc3*) and transcribed in the opposite orientation [95]. Inspection of the density-plots revealed a peak in an intronic region in the *HERC3* locus in *Bos taurus* (Fig. S2). This peak is a CpG island (CpG28) at the 5’ end of *NAP1L5* and thus defines a candidate ICR for imprinted gene expression. Since the peak is at a similar location as the one observed in the known *Nap1l5* ICR in mouse, our strategy accurately pinpointed the *NAP1L5* ICR in cattle.

Noteworthy could be that in evaluations of plots, we also observed a peak in a CpG island at the 5’ end of *DGAT1* (Fig. S3). This finding predicted that *DGAT1* is an imprinted gene. Consistent with this interpretation is that in cattle, *DGAT1* is expressed from the maternal allele [2]. In metabolic pathways, diacylglycerol acyltransferase 1 (DGAT1) catalyzes the last step in triacylglycerol synthesis in the mammary gland. Therefore, *DGAT1* underlies genetic variations in milk-fat composition of dairy cows [96]. In cattle, buffalo, goat, and sheep, *DGAT1* is associated not only with milk but also with meat characteristics [97]. Furthermore, *DGAT1* was positively selected amongst European *Bos taurus* breed with large phenotypic effects [98].

### Within *Bos taurus* chromosome 26, a peak predicted a candidate ICR for imprinted *INPP5F_V2* expression

INPP5F is an inositol 4-phosphatase that functions in the endocytic pathway [99]. In mice, *Inpp5f_v2* was identified as an imprinted gene in the brain [100]. *Inpp5f_v2* is a variant of *Inpp5f*. It is a retrogene with a unique alternative first exon [100]. *Inpp5f_v2* transcription originates in a CpG island within intron 15 in *Inpp5f*  [100]. However, in literature surveys, we could not find any report concerning cattle *INPPF5_V2*. In mouse, *Inpp5f* is biallelically expressed; *Inpp5f_v2* is transcribed from the paternal allele [100]. In that *locus*, CpG analyses identified two CpG islands [100]. CpG1 is near the 5′ end of *Inpp5f*, CpG2 is at 5′ end of *Inpp5f_v2*. Bisulfite sequencing of CpG1 showed that both *Inpp5f* alleles were hypomethylated, as would be expected for a nonimprinted transcriptionally active gene. In mouse brain, CpG2 was methylated on *Inpp5f_v2* maternal allele, but not on paternal allele [100]. Thus, the intragenic island regulates imprinted *Inpp5f_v2* expression [100]. Similarly, the promotor of human *INPPF5_V2* transcript is embedded within a maternally methylated DMR [101].

Even though in literature surveys, we could not find any report concerning cattle *INPPF5_V2*, we imagined that our strategy may provide clues into imprinted expression of this retrogene in *Bos taurus*.

**Fig. 10:**
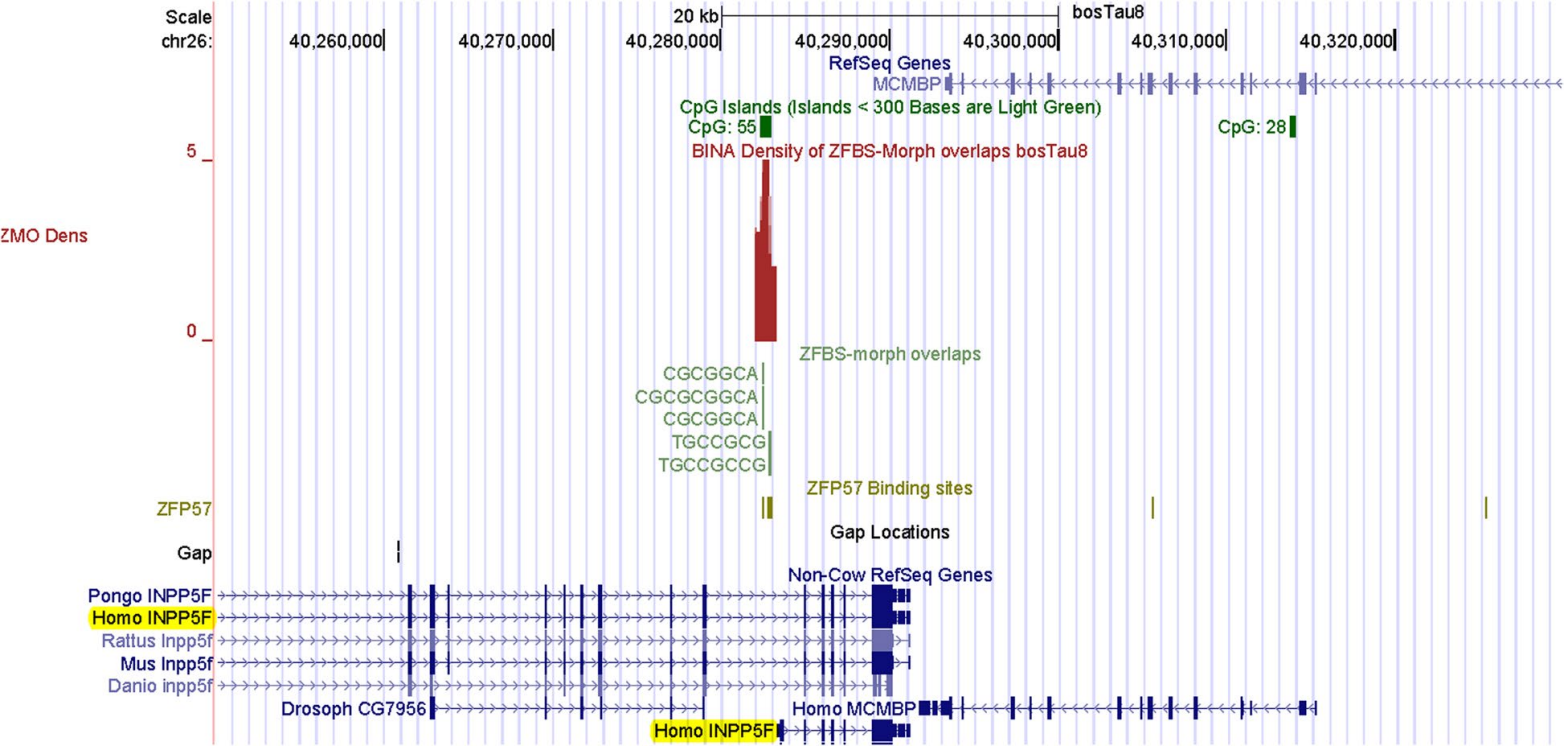
With respect to Non-Cow RefSeq Genes, a robust-peak predicting the ICR for imprinted expression of *INPP5F_V2* in cattle. As observed for mice and humans, this peak is in an intragenic CpG island upstream *MCMBP*

However, the genome browser did not include any annotation for the *INPPF5* in the build bosTau8. Thus, we were dealing with a blank canvas to explore whether or not our strategy could locate a candidate ICR in the absence of complete information. To explore this idea, we located *INPP5F* transcripts in the context of Non-Cow RefSeq Genes. With respect to human transcripts, our approach revealed a candidate ICR for imprinted expression of *INPPF5_V2* in cattle (Fig. 10). This candidate maps to a CpG island (CpG55) upstream of *MCMBP*. In both human and mouse, this gene is downstream of *INPPF5*. Thus, we were in correct genomic area in cattle (Fig. 10). With respect to human transcripts, the candidate ICR is at the 5’ end of *INPPF5_V2*. Furthermore, with respect to human transcripts, CpG55 in cattle is intragenic. Thus, as demonstrated for mouse (Additional file 1), our strategy could find an intragenic ICR for imprinted expression of *INPPF5_V2* in cattle (Fig. 10).

### The density-plots could facilitate locating candidate ICRs in *Bos taurus*

**Fig. 11:**
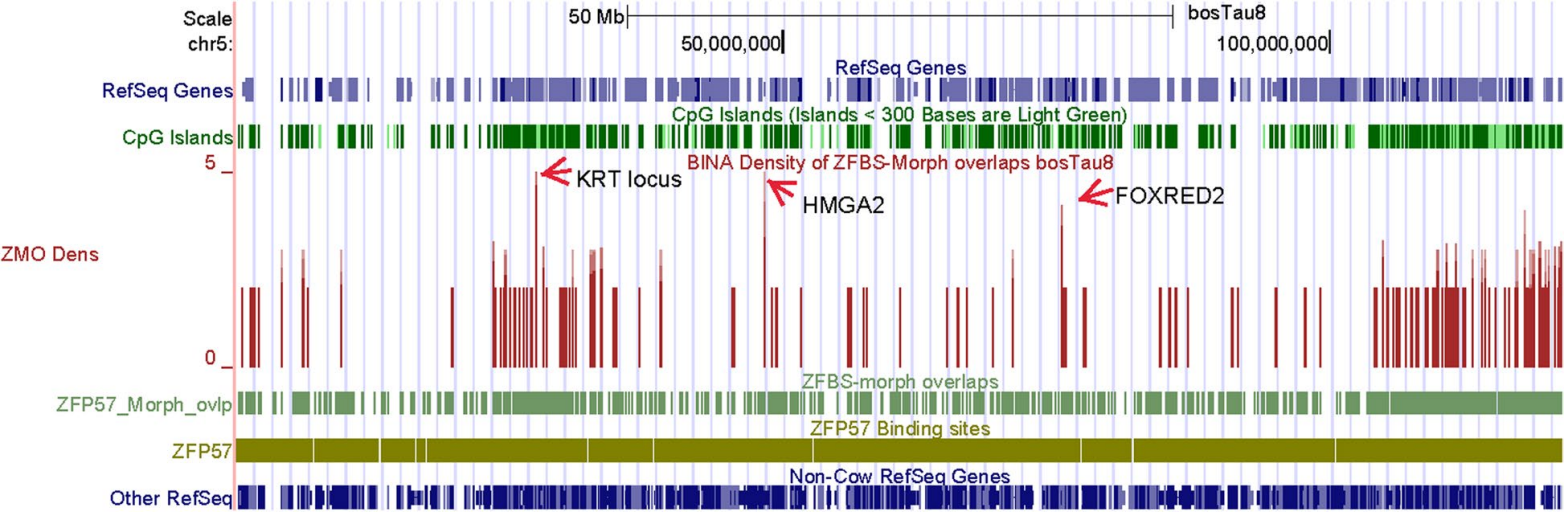
A snapshot of the density-plot obtained for the entire Chr5. Three very robust peaks point to candidate ICRs predicting imprinted gene expression from *KRT*, *HMGA2*, *FOXRED2 loci*. Peaks covering 2 ZFBS-morph overlaps could be true or false-positives

Since the density-plots were created genome-wide, we could view peak positions along the DNA in an entire chromosome. For example, examine a snapshot from the UCSC genome browser presenting peak positions along the entire DNA in Chr5 (Fig. 11). In the build bosTau8, this chromosome covers greater than 121,2 Mb DNA. We find that even along an entire chromosome, many peaks are clearly or almost fully resolved. This and related findings demonstrate that peaks in plots occur infrequently in cattle DNA. This outcome is expected because CpG frequency is relatively low in animal DNA [102]. Since peaks in plots encompass several CpGs, they represent uncommon events along genomic DNA. Additionally, as one would expect, peaks covering 3 or more ZFBS-morph overlaps are sparser than those that cover 2 (Fig. 11).

**Fig. 12:**
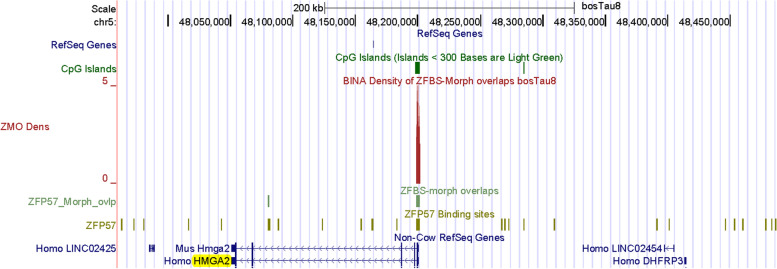
A closeup view of a candidate ICR for imprinted expression of *HMGA2*. The corresponding peak is in a CpG island

**Fig. 13:**
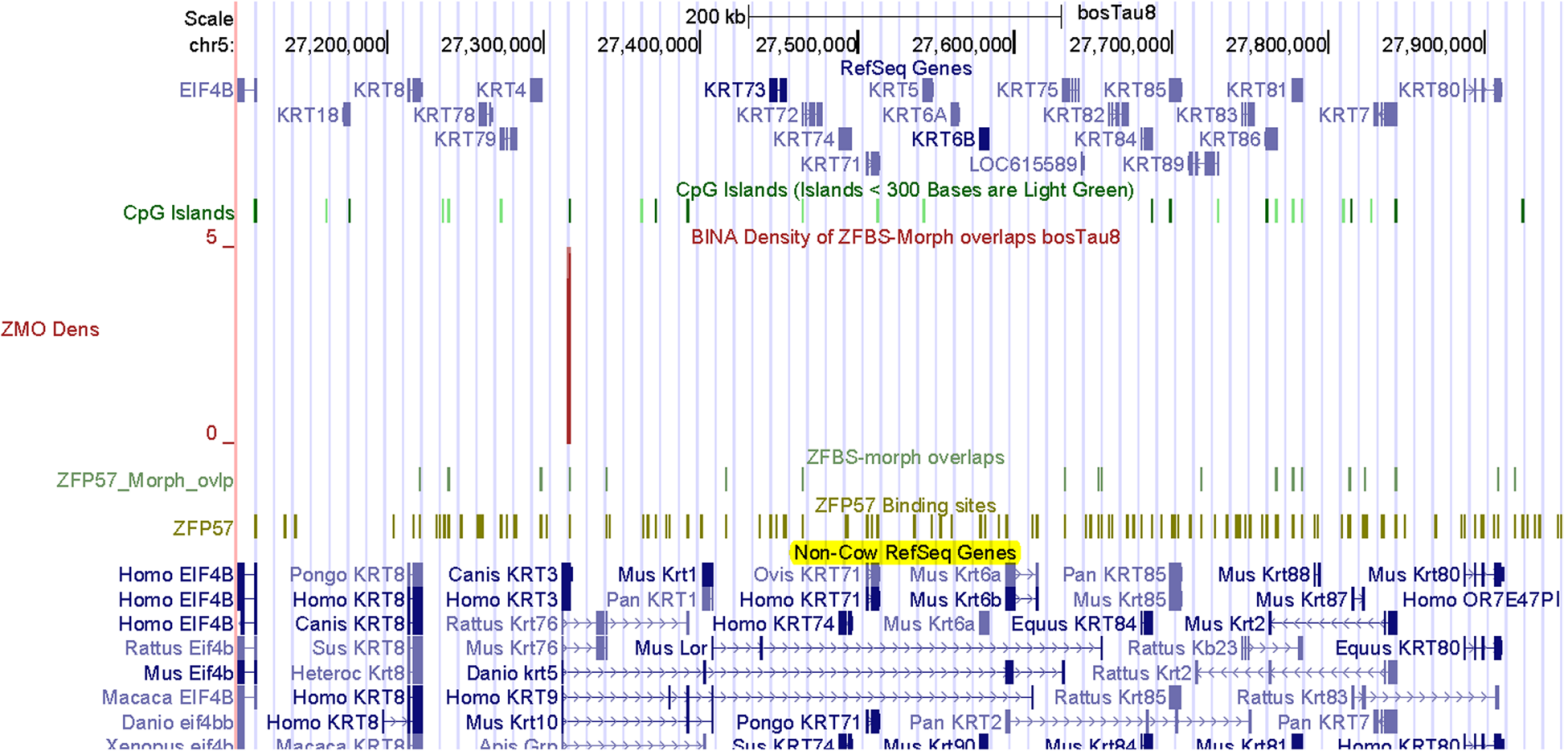
A candidate ICR in the *KRT* locus. The corresponding peak is in a CpG island. Although the peak is upstream of *KRT4* in cattle, the predicted ICR might be regulating imprinted expression of an unannotated gene in *Bos taurus* genomic DNA

**Fig. 14:**
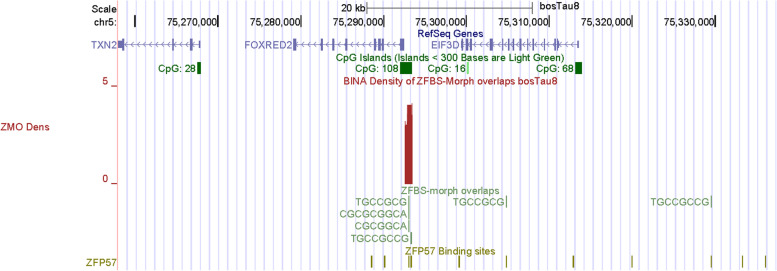
A closeup view of a candidate ICR for imprinted expression of *FOXRED2*. The peak is in a CpG island

Since in plots, several peaks correctly identified regions that include known ICRs and inferred DMRs (Figs. 1, 2, 3, 4, 5, 6, 7, 8, 9 and 10), we imagined that additional peaks may correspond to candidate ICRs. Along Chr5, examples include 3 very robust peaks mapping to *HMGA2*, *KRT*, and *FOXRED2 loci* (Fig. 11). On the genome browser, we could obtain enlarged views to inspect peak positions with respect to nearby genes (Figs. 12, 13 and 14). Along Chr5, one of the 3 very robust peaks maps to a CpG island in an unannotated region in *Bos taurus*. With respect to Non-Cow RefSeq Genes, the peak is at the 5’ end of *HMGA2* (Fig. 12). HMGA2 belongs to a superfamily of nuclear proteins [103]. Their structure includes a domain (AT-Hook) that binds DNA and nucleosomes with no strong preference for the underlying DNA sequence [103]. Polymorphisms in *HMGA2* affected height in human and body stature in cattle [104]. This gene also impacts body size in mice [105], rabbits [106], horses [107], Shetland ponies and other small horses [108]. By applying genome-wide association studies for cattle stature, meta-analysis identified common genes that impacted body size [109]. The gene list included *HMGA2*. The association studies involved 58,265 cattle from 17 populations with 25.4 million imputed whole-genome sequence variants.

**Fig. 15:**
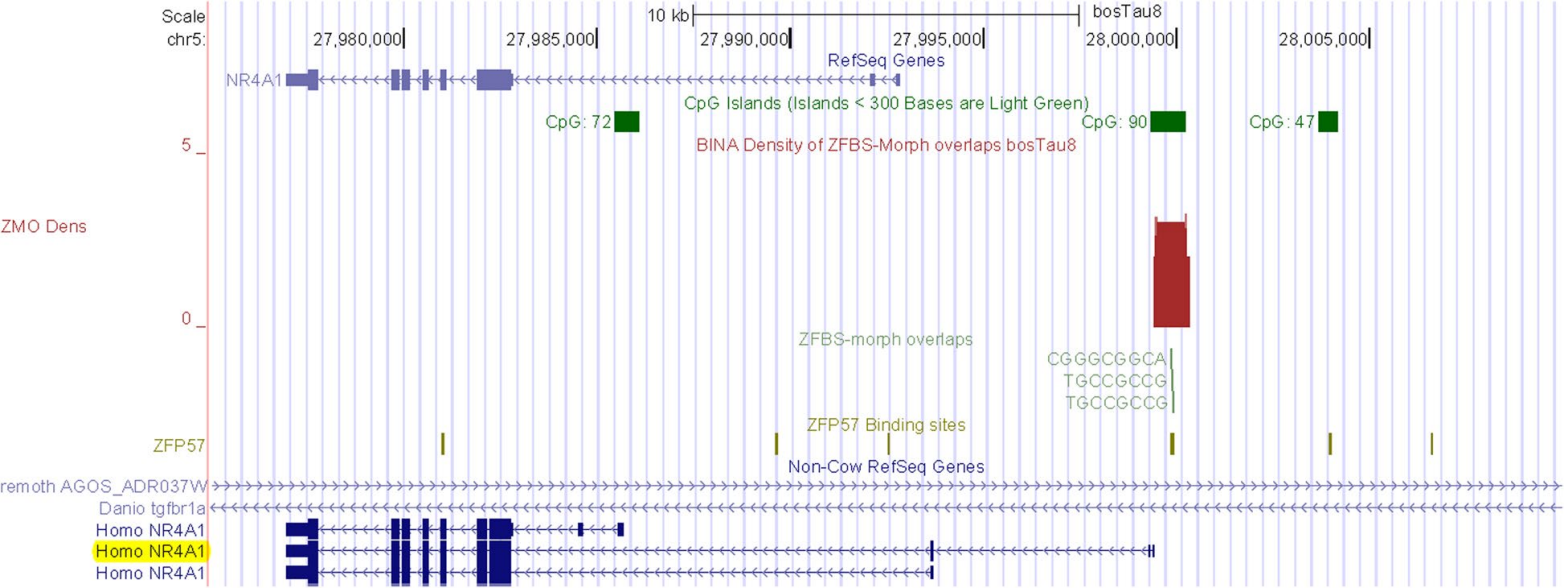
A candidate ICR regulating predicted imprinted *NR4A1* expression. The corresponding peak maps to a CpG island

Along Chr5, another very robust peak maps to *KRT* (keratin) *locus* (Fig. 13). Keratin is one of the most important structural proteins in nature and is widely found in the integument in vertebrates [110]. After collagen, it is the most important biopolymer encountered in animals. In cow, sheep, goat, and pig, keratin is a constituent of skin, fur, wool, and hoof [110]. As other animal genomes, cattle DNA includes a DNA segment encompassing a cluster of *KRT* genes (Fig. 13). In the density-plot of a large section of Chr5, we noticed a robust peak mapping to a CpG island downstream of *KRT4*. However, when examined in the context of Non-Cow RefSeq Genes, it seems that the island maps to a *KRT* gene that is not annotated in *Bos taurus* DNA (Fig. 13). Along Chr5, another very robust peak maps to a CpG island that encompasses the promotor of *FOXRED2* (Fig. 14). Also known as ERFAD, FOXRED2 is a luminal flavoprotein that functions in endoplasmic reticulum-associated degradation [111]. It facilitates the dislocation of certain endoplasmic reticulum-associated substrates to the cytosol. Although not in the very robust category, another peak in Chr5 maps to *NR4A1* (Fig. 15). Nonetheless, the peak is robust since it encompasses 3 ZFBS-morph overlaps. It maps to the 5’ end of one of human *NR4A1* transcriptional isoforms. For display we chose *NR4A1* since it was among the candidate genes potentially driving tissue-specific differences between genetically distinct subspecies of cattle arising from independent domestication events [112].

**Fig. 16:**
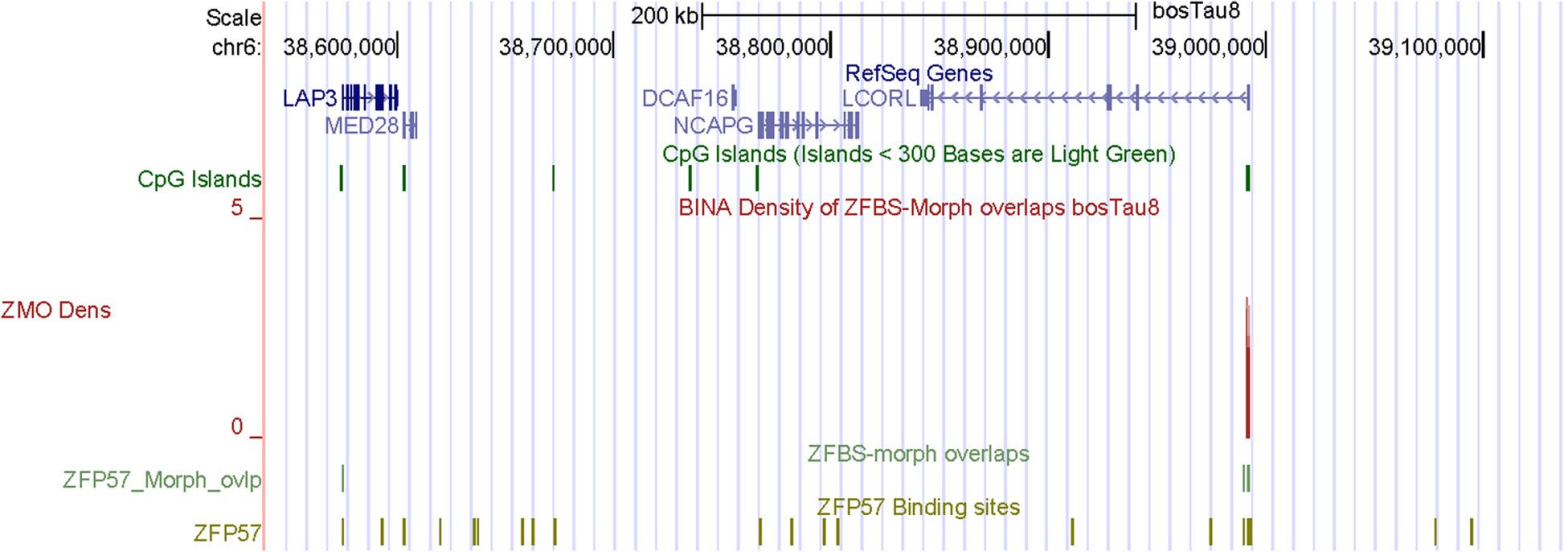
A closeup view of a candidate ICR for imprinted expression of *LCORL*. The corresponding peak maps to a CpG island

In the context of body stature, in addition to *HMGA2* we came across *LCORL*. The density-plots include a very robust peak in the CpG island at the *LCORL* promotor (Fig. 16). Polymorphisms in *LCORL* are associated with stature in dog [113], horse [107, 114], pig [115], and sheep [116, 117]. In cattle, *LCORL* is in a chromosomal section that includes *NCAPG* (Fig. 16). In genome-wide association studies of cattle stature, *LCORL* and *NCAPG* were among the genes regulating body size [109]. Furthermore, both *LCORL* and *NCAPG* were associated with *loci* that underwent selective sweep in *Bos taurus* populations [98]. In beef cattle, both *loci* influence feed intake, meat, and carcass traits [3]. Functionally, *LCOR* and *LCORL* encode proteins that balance PRC2 subtype activities [118]. In higher eukaryotes, Polycomb group complexes (PRC) are essential for maintaining cellular identity [119].

While *LCOR* and *LCORL* impact body stature, *PTEN* influences milk production [120]. With our strategy, we identified *PTEN* as a potential imprinted gene regulated by a candidate ICR in Chr6 (Fig. S4). This predicted ICR is in a CpG island that encompasses the *PTEN* promoter, TSS, and exon 1. The PTEN-AKT pathway functions in the initiation of lactation through the induction of autocrine prolactin [121]. In dairy cows, PTEN inhibited mammary gland development and lactation [120]. In mammary epithelial cells, PTEN down-regulated secretion of beta-casein, triglyceride, and lactose. Thus, PTEN played a critical role in lactation related signaling pathways in dairy cows [120]. Noteworthy could be that in European cattle breeds, *PTEN* was among the genes in breed-specific selection sweeps [98].

While sampling peak positions in plots, we discovered additional candidate imprinting genes (Table 2). The listing includes *SIX1*. The gene product affects eye formation [122]. In mice, *Six1* directly regulated the expression of gamma-crystallin genes and was essential for lens development [123]. *Six1* also impacted myogenesis [124]. Mice lacking *Six1* died at birth because of severe rib malformations and showed extensive muscle hypoplasia affecting most of the body muscles in particular certain hypaxial muscles [124]. Furthermore, myogenesis was altered in *Six1*-deficient mice [124]. Since *Six1* impacted *MyoD* and *myogenin* expression, it was required for primary myogenesis of most body muscles, particularly those of hypaxial origin [124]. In *Six1*-deficient mice, additional processes were affected: including, development of the inner ear, nose, thymus, and kidney [125].

**Table 2:** A sample of candidate imprinted genes in *Bos taurus*

Cattle	Gene	Full name
chr1	*ZBTB21*	zinc finger and BTB domain containing 21
chr1	*BCL6*	BCL6 transcription repressor
Chr3	*POU3F1*	POU class 3 homeobox 1
chr4	*LCORL*	ligand dependent nuclear receptor corepressor
chr4	*BBS9*	Bardet-Biedl syndrome 9
chr5	*HMGA2*	high mobility group AT-hook 2
chr5	*GTSE1*	G2 and S-phase expressed 1
chr6	*ANKRD17*	ankyrin repeat domain 17
chr7	*HCN4*	hyperpolarization activated cyclic nucleotide gated
chr9	*CNR1*	cannabinoid receptor 1
chr9	*SOBP*	sine oculis binding protein homolog
chr10	*SIX1*	SIX homeobox 1
chr18	*TOMM40*	translocase of outer mitochondrial membrane
chr19	*UNK*	unk zinc finger
chr21	*GABRA5*	gamma-aminobutyric acid type A receptor subunit alpha5
Chr26	*PTEN*	phosphatase and tensin homolog
Chr26	*SUFU*	SUFU negative regulator of hedgehog signaling
chr26	*CNNM1*	cyclin and CBS domain divalent metal cation transport mediator 1
chr26	*TM9SF3*	transmembrane 9 superfamily member 3
Chr29	*RPLP2*	ribosomal protein lateral stalk subunit P2

Along chromosomal DNA, robust peaks were randomly selected to identify the genes in their vicinity. According to the strategy: peaks correspond to candidate ICRs; their nearby genes to potential imprinted genes

Besides *SIX1*, Table 2 lists *SUFU*. In adult mouse testis, SUFU was prominent in elongating spermatids –indicating a role for Hedgehog signaling in spermatogenesis [126]. Furthermore, *SUFU* was among the genes in multi-trait meta-analysis identifying genomic regions associated with sexual precocity in tropical beef cattle [127]. Table 2 includes another gene (*CNNM1*) that also impacts spermatogenesis. In mouse testis, expression of *Cnnm1* was associated with cell cycle and differentiation of spermatogenic cells [128]. Listed in Table 2 is a gene (*CNR1*) that encodes a receptor for tetrahydrocannabinol (the principal component of marijuana). Although prevailing studies emphasize endocannabinoid activity in the brain, this compound also affects reproductive system in males [129].

### Summary

Peaks in the density-plots correctly found 18 of the 20 fully characterized ICRs/gDMRs in mouse (Table 1). In the *Bos taurus* genome, peaks in plots also pinpointed nearly all known or inferred DMRs that functioned in allele-specific gene expression (Table 1). Thus, overall, the density-plots accurately identified a substantial fraction of characterized or inferred ICRs/gDMRs in mouse, human, and *Bos taurus* genomic DNA sequences (Table 1). Additionally, a peak in plots found a candidate ICR in the *DGAT1 locus* (Fig. S3). This finding predicted that *DGAT1* is a potential imprinted gene in cattle. Since *DGAT1* was expressed from the maternal allele [2], the strategy made a correct prediction. Importantly, in cattle *DGAT1* impacted milk and meat characteristics [2, 97].

Furthermore –as previously demonstrated for human and mouse [40, 41]– peaks in plots of entire chromosomal DNA predicted ICRs for many candidates imprinted genes in *Bos taurus* (Fig. 11). Examples include *PTEN*, *HMGA2*, and *LCORL* (Figs. 12, 16, and S4). In dairy cows, PTEN inhibited mammary gland development and lactation [120]. Both *HMGA2* and *LCORL* affected body stature in cattle [104, 109]. Therefore, the predicted ICRs for *HMGA2* and *LCORL* may contribute to studies of LOS. Table 2 lists additional candidate imprinting genes discovered by our approach.

## Discussion

In the course of many centuries, farmers have selected animals with economically and nutritionally important traits including size, meat quality, and milk production [1, 2]. And, literature surveys have identified numerous selection sweeps across 37 cattle breeds [98]. To propagate cattle with desired traits, researchers have also explored assisted reproductive technologies [8, 12, 130]. However, evidence indicates that animals made from cloning and other assisted reproductive tools often display phenotypes of imprinting disruptions [2, 11, 13, 14, 43]. Because of the impact of genomic imprinting on producing normal calves, it is necessary to develop strategies to discover candidate ICRs regulating expression of novel imprinted genes.

Historically, studies of mouse have offered a rich source of data for identifying imprinting genes in other species including cattle [2]. Previously, we applied our predictive strategy to mouse and uncovered several candidate ICRs in vicinity of genes that they may control [40]. In studies of the human genome, our strategy predicted ICRs for parent-of-origin specific expression of several experimentally inferred imprinted genes [41]. In human DNA, the density-plots also located candidate ICRs for potential imprinted genes associated with disease-states and developmental anomalies know as syndromes. Examples include association of: *ARID1B* with Coffin-Siris syndrome; *PCNT* with microcephalic osteodysplastic primordial dwarfism type II; *IMPDH1* with Leber congenital amaurosis 11; *PRDM8* with progressive myoclonic epilepsy-10; *CITED2* with ventricular septal defect 2; and *VAX1* with microphthalmia, cleft lip and palate, and agenesis of the corpus callosum [41]. Clearly, it could be challenging to discover such candidate imprinted genes by conventional techniques.

In order to investigate the suitability of our predictive strategy to studies of another mammal, we chose *Bos taurus*. We selected that species, because of the impact of genomic imprinting on the development of normal calves. Furthermore, much is known about: developmental irregularities; epigenetic anomalies; and aberrant DNA methylation imprints in aborted clones [8, 10–13]. We envisage that our predictive strategy could offer the opportunity to investigate genetic variations among cattle breeds in the context of candidate ICRs and imprinted genes.

Our strategy facilitates ICR detection across relatively long genomic DNA sections [40, 41], and even across an entire chromosomal DNA (Fig. 11). In evaluations, we found that peaks in the density-plots, pinpointed known ICRs or putative DMRs in cattle (Table 1). Examples include localization of the ICRs regulating expression of: imprinted domains (Figs. 1, 2, 4 and 6); and intragenic genes and transcripts (Figs. 7, and 8). Furthermore, our strategy predicted the central position of the ICR in *H19*—*IGF2* imprinted domain (Fig. 1) and the essential ICR in the complex *GNAS locus* (Fig. 5). It also predicted candidate ICRs in unannotated or not fully annotated *loci* in the build bosTau8 of *Bos taurus*. Examples include the *PLAGL1, GNAS*, and *INPP5F loc*i (Figs. 3, 5 and 10). Moreover, peaks in plots predicted candidate ICRs for allele-specific expression of *MEG8*, *SNRPN*, and *NAP1L5* (Figs. 9, S1, S2) –known imprinted genes in cattle [9, 92, 93, 131]. Also, a peak in plots found a candidate ICR in the *DGAT1 locus* (Fig. S3). In cattle, *DGAT1* is expressed from the maternal allele [2]. Thus, our strategy recognized *DGAT1* as an imprinted gene. Since DGAT1 catalyzes the last step in triacylglycerol synthesis, it impacts the quality of meat and dairy products. Hence, it is not surprising that *DGAT1* is important to milk and meat characteristics [2, 97]. Mostly, genetic variations in *DGAT1* influenced milk-fat composition of dairy cows [96].

Based on described findings, we imagined that the density-plots could help with the discovery of candidate ICRs for potential novel imprinted genes in *Bos taurus* (Table 2). To explore this idea, one could inspect plots of an entire chromosomal DNA sequence to identify robust peaks defining known or candidate ICRs (Fig. 11). In enlarged views, one could identify genes in the vicinity of candidate ICRs and thus locate potential imprinted genes for experimental validations.

Along Chr26, covering nearly 52 Mb DNA, many of the robust peaks were clearly or almost fully resolved [132]. Similarly, along Chr5 –which encompasses nearly 121,2 Mb DNA– several peaks were fully resolved (Fig. 11). Thus, overall, inspection of plots revealed that robust peaks occurred infrequently along chromosomal DNA. Advantages of our predictive strategy include locating candidate ICRs and imprinted genes not easily detectable or costly to find by conventional techniques. Examples include candidate ICRs in *KRT*, *HMGA2*, and *LCORL loci* (Figs. 12, 13, and 16). In cattle, the *KRT locus* encompasses many genes (Fig. 13). In cow, sheep, goat, and pig, keratin (KRT) is a constituent of skin, fur, wool, and hoof [92]. In meta-analysis, both *HMGA2* and *LCORL* were associated with cattle stature [109]. *Hmga2* knockout mice exhibited impaired muscle development and reduced myoblast proliferation; its overexpression promoted myoblast growth [133]. This finding revealed a possible role for HMGA2 in meat quality. *LCORL* is among the genes regulating body size [109]. Furthermore, in *Bos taurus* populations, the *LCORL* locus has undergone selective sweeps [98]. Considering the importance of genomic imprinting in normal growth and developmental processes in cattle [2, 11, 13, 14, 43], it might be significant that our strategy identified *HMGA2* and *LCORL* as candidate imprinted genes and ICRs for their allele-specific expression (Figs. 12 and 16). Furthermore, the density plots revealed a candidate ICR for imprinted expression of *PTEN* (Fig. S4). While *LCOR* and *LCORL* impact body stature, *PTEN* influences milk production [120]. Table 2 lists additional candidate imprinted genes. They were discovered while we were sampling genes in the vicinity of peaks, in order to explore the power of our approach.

## Conclusion

In this report, we offered a predictive genome-wide strategy to discover candidate ICRs and novel imprinted genes in *Bos taurus*. We gave evidence for robustness of our strategy by pinpointing several of the well-known ICRs and inferred DMRs in various chromosomal DNA sections. We also showed discovery of candidate ICRs for known imprinted genes and transcripts in unannotated or not fully annotated segments in *Bos taurus* genomic DNA. We also gave examples of how with our strategy, one could view the positions of known and candidate ICRs along the entire chromosomal DNA sequences. If surveyed in the context of the wealth of known genetic variations among cattle breeds, our strategy could serve as a resource for locating candidate ICRs and their nearby genes. Clearly, predictive methods require experimental validations. Therefore, below we offer links for downloading our datasets for creating custom tracks on the UCSC genome browser. The browser offers a great resource for studies of genomic DNA sequences in higher organisms [134–136]. On the browser, default tracks facilitate viewing our datasets in the context of genomic landmarks including genes, transcripts, the CpG islands, SNPs, and much more [137].

## Methods

### Marking the genomic positions of ZFP57 binding site and the ZFBS-morph overlaps

For studies of genomic imprinting in *Bos taurus*, we followed a previous approach applied to mouse and human genomic DNA [40, 41]. Briefly: at the UCSC genome browser, we retrieved the DNA sequences of cattle chromosomes (reported for the build bosTau8). Next, we wrote a Perl script to obtain the genomic positions of the reported hexameric ZFP57 binding site [21], and the positions of ZFBS-morph overlaps [60]. The script opened the file containing the nucleotide sequence of a specified chromosome, as well as either the file containing the ZFP57 binding site or sequences of the ZFBS-morph overlaps. Subsequently, it located their genomic positions. With UNIX subroutines, we combined the outputs obtained for various chromosomes to create a file suitable for upload onto the UCSC genome browser.

### Creating plots of the density of ZFBS-morph overlaps in genomic DNA

With another Perl script, we established the genomic positions of DNA segments that covered 2 or more closely spaced ZFBS morph overlaps. That script opened the file containing the positions of ZFBS-morph overlaps for a specified chromosome. Subsequently, the script scanned the file to count and report the number of ZFBS-morph overlaps within a sliding window consisting of 850-bases. We selected the window size by trial and error. Large windows tended to produce false peaks; small windows gave peaks with a spiky appearance. To remove background noise, the script ignored isolated overlaps. Next, with a UNIX subroutine, we combined and tailored the outputs of the program for display as a custom track on the UCSC genome browser. While creating our datasets, bosTau8 was the latest available build.

### Localization of predicted CTCF sites

We found predicted CTCF sites using a server at the University of Tennessee Health Science Center [138]. We chose that server because previously it correctly predicted CTCF binding sites in the ICR of human *H19*—*IGF2* imprinted domain [139]. The positions of predicted sites agreed with results of ChIPs reporting the association of a subset of nuclear proteins (*i.e*., CTCF, RAD21, and SMC3) with chromatin [140].

## Supplementary Information

**Additional file 1.**

**Additional file 2.**

## Data Availability

Via the links below, you can access the datasets for download: - Positions of ZFBS and ZFBS-morph overlaps in the build bosTau8 of the *Bos taurus* genome https://purr.purdue.edu/publications/3359/1 - The density of ZFBS-morph overlaps in the build bosTau8 https://purr.purdue.edu/publications/3360/1

## Abbreviations

PRC2: Polycomb repressive complexes 2
morph: MLL morpheme
TSS: Transciption start site
ZFBS: ZFP57 binding site

## References

[1] Magee DA, Spillane C, Berkowicz EW, Sikora KM, MacHugh DE (2014). Imprinted loci in domestic livestock species as epigenomic targets for artificial selection of complex traits. Anim Genet.

[2] Tian XC (2014). Genomic imprinting in farm animals. Annu Rev Anim Biosci.

[3] Lindholm-Perry AK, Sexten AK, Kuehn LA, Smith TP, King DA, Shackelford SD, Wheeler TL, Ferrell CL, Jenkins TG, Snelling WM (2011). Association, effects and validation of polymorphisms within the NCAPG - LCORL locus located on BTA6 with feed intake, gain, meat and carcass traits in beef cattle. BMC Genet.

[4] Berry DP, Wall E, Pryce JE (2014). Genetics and genomics of reproductive performance in dairy and beef cattle. Animal.

[5] Rezende FM, Dietsch GO, Penagaricano F (2018). Genetic dissection of bull fertility in US Jersey dairy cattle. Anim Genet.

[6] Micke GC, Sullivan TM, McMillen IC, Gentili S, Perry VE (2011). Protein intake during gestation affects postnatal bovine skeletal muscle growth and relative expression of IGF1, IGF1R, IGF2 and IGF2R. Mol Cell Endocrinol.

[7] Kagitani F, Kuroiwa Y, Wakana S, Shiroishi T, Miyoshi N, Kobayashi S, Nishida M, Kohda T, Kaneko-Ishino T, Ishino F (1997). Peg5/Neuronatin is an imprinted gene located on sub-distal chromosome 2 in the mouse. Nucleic Acids Res.

[8] Chen Z, Hagen DE, Elsik CG, Ji T, Morris CJ, Moon LE, Rivera RM (2015). Characterization of global loss of imprinting in fetal overgrowth syndrome induced by assisted reproduction. Proc Natl Acad Sci U S A.

[9] Smith LC, Therrien J, Filion F, Bressan F, Meirelles FV (2015). Epigenetic consequences of artificial reproductive technologies to the bovine imprinted genes SNRPN, H19/IGF2, and IGF2R. Front Genet.

[10] Liu JH, Yin S, Xiong B, Hou Y, Chen DY, Sun QY (2008). Aberrant DNA methylation imprints in aborted bovine clones. Mol Reprod Dev.

[11] Smith LC, Suzuki J, Jr., Goff AK, Filion F, Therrien J, Murphy BD, Kohan-Ghadr HR, Lefebvre R, Brisville AC, Buczinski S et al. Developmental and epigenetic anomalies in cloned cattle. Reprod Domestic Anim. 2012;47(Suppl 4):107–114. 10.1111/j.1439-0531.2012.02063.x 22827358

[12] Urrego R, Rodriguez-Osorio N, Niemann H (2014). Epigenetic disorders and altered gene expression after use of Assisted Reproductive Technologies in domestic cattle. Epigenetics.

[13] O'Doherty AM, MacHugh DE, Spillane C, Magee DA (2015). Genomic imprinting effects on complex traits in domesticated animal species. Front Genet.

[14] O'Doherty AM, McGettigan P, Irwin RE, Magee DA, Gagne D, Fournier E, Al-Naib A, Sirard MA, Walsh CP, Robert C (2018). Intragenic sequences in the trophectoderm harbour the greatest proportion of methylation errors in day 17 bovine conceptuses generated using assisted reproductive technologies. BMC Genomics.

[15] Li Y, Donnelly CG, Rivera RM (2019). Overgrowth Syndrome. Vet Clin North Am Food Anim Pract.

[16] Strogantsev R, Krueger F, Yamazawa K, Shi H, Gould P, Goldman-Roberts M, McEwen K, Sun B, Pedersen R, Ferguson-Smith AC (2015). Allele-specific binding of ZFP57 in the epigenetic regulation of imprinted and non-imprinted monoallelic expression. Genome Biol.

[17] Stewart KR, Veselovska L, Kelsey G (2016). Establishment and functions of DNA methylation in the germline. Epigenomics.

[18] Barlow DP, Bartolomei MS: Genomic imprinting in mammals. Cold Spring Harbor perspectives in biology 2014, 6(2). 10.1101/cshperspect.a018382 PMC3941233 24492710

[19] Bartolomei MS, Tilghman SM (1997). Genomic imprinting in mammals. Annu Rev Genet.

[20] Proudhon C, Bourc'his D (2010). Evolution of genomic imprinting in mammals: what a zoo!. Med Sci (Paris).

[21] Quenneville S, Verde G, Corsinotti A, Kapopoulou A, Jakobsson J, Offner S, Baglivo I, Pedone PV, Grimaldi G, Riccio A (2011). In embryonic stem cells, ZFP57/KAP1 recognize a methylated hexanucleotide to affect chromatin and DNA methylation of imprinting control regions. Mol Cell.

[22] Strogantsev R, Ferguson-Smith AC (2012). Proteins involved in establishment and maintenance of imprinted methylation marks. Brief Funct Genomics.

[23] Helleboid PY, Heusel M, Duc J, Piot C, Thorball CW, Coluccio A, Pontis J, Imbeault M, Turelli P, Aebersold R (2019). The interactome of KRAB zinc finger proteins reveals the evolutionary history of their functional diversification. EMBO J.

[24] Huntley S, Baggott DM, Hamilton AT, Tran-Gyamfi M, Yang S, Kim J, Gordon L, Branscomb E, Stubbs L (2006). A comprehensive catalog of human KRAB-associated zinc finger genes: insights into the evolutionary history of a large family of transcriptional repressors. Genome Res.

[25] Riso V, Cammisa M, Kukreja H, Anvar Z, Verde G, Sparago A, Acurzio B, Lad S, Lonardo E, Sankar A (2016). ZFP57 maintains the parent-of-origin-specific expression of the imprinted genes and differentially affects non-imprinted targets in mouse embryonic stem cells. Nucleic Acids Res.

[26] Bina M (2017). Imprinted control regions include composite DNA elements consisting of the ZFP57 binding site overlapping MLL1 morphemes. Genomics.

[27] Bina M, Wyss P, Novorolsky E, Zulkelfi N, Xue J, Price R, Fay M, Gutmann Z, Fogler B, Wang D (2013). Discovery of MLL1 binding units, their localization to CpG Islands, and their potential function in mitotic chromatin. BMC Genomics.

[28] Bina M, Wyss P (2015). Impact of the MLL1 morphemes on codon utilization and preservation in CpG Islands. Biopolymers.

[29] Bina M, Wyss P, Ren W, Szpankowski W, Thomas E, Randhawa R, Reddy S, John PM, Pares-Matos EI, Stein A (2004). Exploring the characteristics of sequence elements in proximal promoters of human genes. Genomics.

[30] Bina M, Wyss P, Lazarus SA, Shah SR, Ren W, Szpankowski W, Crawford GE, Park SP, Song XC (2009). Discovering sequences with potential regulatory characteristics. Genomics.

[31] Ruthenburg AJ, Allis CD, Wysocka J (2007). Methylation of lysine 4 on histone H3: intricacy of writing and reading a single epigenetic mark. Mol Cell.

[32] Zhou VW, Goren A, Bernstein BE (2011). Charting histone modifications and the functional organization of mammalian genomes. Nat Rev Genet.

[33] Birke M, Schreiner S, Garcia-Cuellar MP, Mahr K, Titgemeyer F, Slany RK (2002). The MT domain of the proto-oncoprotein MLL binds to CpG-containing DNA and discriminates against methylation. Nucleic Acids Res.

[34] Bach C, Mueller D, Buhl S, Garcia-Cuellar MP, Slany RK (2009). Alterations of the CxxC domain preclude oncogenic activation of mixed-lineage leukemia 2. Oncogene.

[35] Ayton PM, Chen EH, Cleary ML (2004). Binding to nonmethylated CpG DNA is essential for target recognition, transactivation, and myeloid transformation by an MLL oncoprotein. Mol Cell Biol.

[36] Jones WD, Dafou D, McEntagart M, Woollard WJ, Elmslie FV, Holder-Espinasse M, Irving M, Saggar AK, Smithson S, Trembath RC (2012). De novo mutations in MLL cause Wiedemann-Steiner syndrome. Am J Hum Genet.

[37] Meyer E, Carss KJ, Rankin J, Nichols JM, Grozeva D, Joseph AP, Mencacci NE, Papandreou A, Ng J, Barral S (2017). Mutations in the histone methyltransferase gene KMT2B cause complex early-onset dystonia. Nat Genet.

[38] Meyer E, Carss KJ, Rankin J, Nichols JME, Grozeva D, Joseph AP, Mencacci NE, Papandreou A, Ng J, Barral S (2017). Corrigendum: Mutations in the histone methyltransferase gene KMT2B cause complex early-onset dystonia. Nat Genet.

[39] Andreu-Vieyra CV, Chen R, Agno JE, Glaser S, Anastassiadis K, Stewart AF, Matzuk MM: MLL2 is required in oocytes for bulk histone 3 lysine 4 trimethylation and transcriptional silencing. PLoS Biol. 2010;8(8). 10.1371/journal.pbio.1000453 PMC2923083 20808952

[40] Bina M, Wyss P: Simultaneous discovery of candidate imprinted genes and Imprinting Control Regions in the mouse genome. bioRxiv 2019.

[41] Bina M (2020). Discovering candidate imprinted genes and imprinting control regions in the human genome. BMC Genomics.

[42] Verona RI, Mann MR, Bartolomei MS (2003). Genomic imprinting: intricacies of epigenetic regulation in clusters. Annu Rev Cell Dev Biol.

[43] Curchoe CL, Zhang S, Yang L, Page R, Tian XC (2009). Hypomethylation trends in the intergenic region of the imprinted IGF2 and H19 genes in cloned cattle. Anim Reprod Sci.

[44] Yang L, Chavatte-Palmer P, Kubota C, O'Neill M, Hoagland T, Renard JP, Taneja M, Yang X, Tian XC (2005). Expression of imprinted genes is aberrant in deceased newborn cloned calves and relatively normal in surviving adult clones. Mol Reprod Dev.

[45] Hori N, Nagai M, Hirayama M, Hirai T, Matsuda K, Hayashi M, Tanaka T, Ozawa T, Horike S (2010). Aberrant CpG methylation of the imprinting control region KvDMR1 detected in assisted reproductive technology-produced calves and pathogenesis of large offspring syndrome. Anim Reprod Sci.

[46] Hansmann T, Heinzmann J, Wrenzycki C, Zechner U, Niemann H, Haaf T (2011). Characterization of differentially methylated regions in 3 bovine imprinted genes: a model for studying human germ-cell and embryo development. Cytogenet Genome Res.

[47] Bartolomei MS (2009). Genomic imprinting: employing and avoiding epigenetic processes. Genes Dev.

[48] Robbins KM, Chen Z, Wells KD, Rivera RM (2012). Expression of KCNQ1OT1, CDKN1C, H19, and PLAGL1 and the methylation patterns at the KvDMR1 and H19/IGF2 imprinting control regions is conserved between human and bovine. J Biomed Sci.

[49] Engemann S, Strodicke M, Paulsen M, Franck O, Reinhardt R, Lane N, Reik W, Walter J (2000). Sequence and functional comparison in the Beckwith-Wiedemann region: implications for a novel imprinting centre and extended imprinting. Hum Mol Genet.

[50] Lewis A, Green K, Dawson C, Redrup L, Huynh KD, Lee JT, Hemberger M, Reik W (2006). Epigenetic dynamics of the Kcnq1 imprinted domain in the early embryo. Development.

[51] Tycko B, Morison IM (2002). Physiological functions of imprinted genes. J Cell Physiol.

[52] Fitzpatrick GV, Soloway PD, Higgins MJ (2002). Regional loss of imprinting and growth deficiency in mice with a targeted deletion of KvDMR1. Nat Genet.

[53] Wang M, Li D, Zhang M, Yang W, Cui Y, Li S (2015). Methylation of KvDMR1 involved in regulating the imprinting of CDKN1C gene in cattle. Anim Genet.

[54] Abdollahi A (2007). LOT1 (ZAC1/PLAGL1) and its family members: mechanisms and functions. J Cell Physiol.

[55] Juma AR, Damdimopoulou PE, Grommen SV, Van de Ven WJ, De Groef B (2016). Emerging role of PLAG1 as a regulator of growth and reproduction. J Endocrinol.

[56] Arima T, Drewell RA, Arney KL, Inoue J, Makita Y, Hata A, Oshimura M, Wake N, Surani MA (2001). A conserved imprinting control region at the HYMAI/ZAC domain is implicated in transient neonatal diabetes mellitus. Hum Mol Genet.

[57] Arima T, Drewell RA, Oshimura M, Wake N, Surani MA (2000). A novel imprinted gene, HYMAI, is located within an imprinted domain on human chromosome 6 containing ZAC. Genomics.

[58] Smith RJ, Arnaud P, Konfortova G, Dean WL, Beechey CV, Kelsey G (2002). The mouse Zac1 locus: basis for imprinting and comparison with human ZAC. Gene.

[59] Arima T, Yamasaki K, John RM, Kato K, Sakumi K, Nakabeppu Y, Wake N, Kono T (2006). The human HYMAI/PLAGL1 differentially methylated region acts as an imprint control region in mice. Genomics.

[60] Bina M, Wyss P, Song XC (2017). Datasets on the genomic positions of the MLL1 morphemes, the ZFP57 binding site, and ZFBS-Morph overlaps in the build mm9 of the mouse genome. Data Brief.

[61] O'Doherty AM, O'Shea LC, Fair T (2012). Bovine DNA methylation imprints are established in an oocyte size-specific manner, which are coordinated with the expression of the DNMT3 family proteins. Biol Reprod.

[62] Braidotti G, Baubec T, Pauler F, Seidl C, Smrzka O, Stricker S, Yotova I, Barlow DP (2004). The Air noncoding RNA: an imprinted cis-silencing transcript. Cold Spring Harb Symp Quant Biol.

[63] Moore GE, Ishida M, Demetriou C, Al-Olabi L, Leon LJ, Thomas AC, Abu-Amero S, Frost JM, Stafford JL, Chaoqun Y (2015). The role and interaction of imprinted genes in human fetal growth. Philos Trans R Soc Lond B Biol Sci.

[64] Stoger R, Kubicka P, Liu CG, Kafri T, Razin A, Cedar H, Barlow DP (1993). Maternal-specific methylation of the imprinted mouse Igf2r locus identifies the expressed locus as carrying the imprinting signal. Cell.

[65] Brown J, Jones EY, Forbes BE (2009). Keeping IGF-II under control: lessons from the IGF-II-IGF2R crystal structure. Trends Biochem Sci.

[66] Chao W, D'Amore PA (2008). IGF2: epigenetic regulation and role in development and disease. Cytokine Growth Factor Rev.

[67] Wutz A, Smrzka OW, Schweifer N, Schellander K, Wagner EF, Barlow DP (1997). Imprinted expression of the Igf2r gene depends on an intronic CpG island. Nature.

[68] Barlow DP, Stoger R, Herrmann BG, Saito K, Schweifer N (1991). The mouse insulin-like growth factor type-2 receptor is imprinted and closely linked to the Tme locus. Nature.

[69] Bastepe M (2007). The GNAS Locus: Quintessential Complex Gene Encoding Gsalpha, XLalphas, and other Imprinted Transcripts. Curr Genomics.

[70] Plagge A, Kelsey G, Germain-Lee EL (2008). Physiological functions of the imprinted Gnas locus and its protein variants Galpha(s) and XLalpha(s) in human and mouse. J Endocrinol.

[71] Seifert R, Wenzel-Seifert K, Lee TW, Gether U, Sanders-Bush E, Kobilka BK. Different effects of Gsalpha splice variants on beta2-adrenoreceptor-mediated signaling. The Beta2-adrenoreceptor coupled to the long splice variant of Gsalpha has properties of a constitutively active receptor. J Biol Chem. 1998;273(18):5109–5116. 9556548

[72] He Q, Zhu Y, Corbin BA, Plagge A, Bastepe M: The G protein alpha subunit variant XLalphas promotes inositol 1,4,5-trisphosphate signaling and mediates the renal actions of parathyroid hormone in vivo. Sci Signal. 2015;8(391):ra84. 10.1126/scisignal.aaa9953 PMC4618482 26307011

[73] Aydin C, Aytan N, Mahon MJ, Tawfeek HA, Kowall NW, Dedeoglu A, Bastepe M (2009). Extralarge XL(alpha)s (XXL(alpha)s), a variant of stimulatory G protein alpha-subunit (Gs(alpha)), is a distinct, membrane-anchored GNAS product that can mimic Gs(alpha). Endocrinology.

[74] Weinstein LS, Yu S, Warner DR, Liu J (2001). Endocrine manifestations of stimulatory G protein alpha-subunit mutations and the role of genomic imprinting. Endocr Rev.

[75] Sikora KM, Magee DA, Berkowicz EW, Berry DP, Howard DJ, Mullen MP, Evans RD, Machugh DE, Spillane C (2011). DNA sequence polymorphisms within the bovine guanine nucleotide-binding protein Gs subunit alpha (Gsalpha)-encoding (GNAS) genomic imprinting domain are associated with performance traits. BMC Genet.

[76] Chen Z, Hagen DE, Wang J, Elsik CG, Ji T, Siqueira LG, Hansen PJ, Rivera RM (2016). Global assessment of imprinted gene expression in the bovine conceptus by next generation sequencing. Epigenetics.

[77] Huang JM, Kim J (2009). DNA methylation analysis of the mammalian PEG3 imprinted domain. Gene.

[78] He H, Kim J (2014). Regulation and function of the peg3 imprinted domain. Genomics & informatics.

[79] Relaix F, Weng X, Marazzi G, Yang E, Copeland N, Jenkins N, Spence SE, Sassoon D (1996). Pw1, a novel zinc finger gene implicated in the myogenic and neuronal lineages. Dev Biol.

[80] Li L, Keverne EB, Aparicio SA, Ishino F, Barton SC, Surani MA (1999). Regulation of maternal behavior and offspring growth by paternally expressed Peg3. Science.

[81] Rault JL, van den Munkhof M, Buisman-Pijlman FTA (2017). Oxytocin as an Indicator of Psychological and Social Well-Being in Domesticated Animals: A Critical review. Front Psychol.

[82] Tveden-Nyborg PY, Alexopoulos NI, Cooney MA, French AJ, Tecirlioglu RT, Holland MK, Thomsen PD, D'Cruz NT (2008). Analysis of the expression of putatively imprinted genes in bovine peri-implantation embryos. Theriogenology.

[83] Kaneko-Ishino T, Kuroiwa Y, Miyoshi N, Kohda T, Suzuki R, Yokoyama M, Viville S, Barton SC, Ishino F, Surani MA (1995). Peg1/Mest imprinted gene on chromosome 6 identified by cDNA subtraction hybridization. Nat Genet.

[84] Lefebvre L, Viville S, Barton SC, Ishino F, Surani MA (1997). Genomic structure and parent-of-origin-specific methylation of Peg1. Hum Mol Genet.

[85] Nakabayashi K, Bentley L, Hitchins MP, Mitsuya K, Meguro M, Minagawa S, Bamforth JS, Stanier P, Preece M, Weksberg R (2002). Identification and characterization of an imprinted antisense RNA (MESTIT1) in the human MEST locus on chromosome 7q32. Hum Mol Genet.

[86] McMinn J, Wei M, Sadovsky Y, Thaker HM, Tycko B (2006). Imprinting of PEG1/MEST isoform 2 in human placenta. Placenta.

[87] Kelsey G, Bodle D, Miller HJ, Beechey CV, Coombes C, Peters J, Williamson CM (1999). Identification of imprinted loci by methylation-sensitive representational difference analysis: application to mouse distal chromosome 2. Genomics.

[88] Evans HK, Wylie AA, Murphy SK, Jirtle RL. The neuronatin gene resides in a "micro-imprinted" domain on human chromosome 20q11.2. Genomics. 2001;77(1–2):99–104. 10.1006/geno.2001.6612 11543638

[89] Zaitoun I, Khatib H (2006). Assessment of genomic imprinting of SLC38A4, NNAT, NAP1L5, and H19 in cattle. BMC Genet.

[90] Charlier C, Segers K, Wagenaar D, Karim L, Berghmans S, Jaillon O, Shay T, Weissenbach J, Cockett N, Gyapay G (2001). Human-ovine comparative sequencing of a 250-kb imprinted domain encompassing the callipyge (clpg) locus and identification of six imprinted transcripts: DLK1, DAT, GTL2, PEG11, antiPEG11, and MEG8. Genome Res.

[91] Wang X, Lan X, Radunz AE, Khatib H (2015). Maternal nutrition during pregnancy is associated with differential expression of imprinted genes and DNA methyltranfereases in muscle of beef cattle offspring. J Anim Sci.

[92] Hou XH, Li DJ, Su H, Hu JQ, Li N, Li SJ (2011). Molecular cloning, expression, and imprinting status of maternally expressed gene 8 (Meg8) in dairy cattle. Genetika.

[93] Lucifero D, Suzuki J, Bordignon V, Martel J, Vigneault C, Therrien J, Filion F, Smith LC, Trasler JM (2006). Bovine SNRPN methylation imprint in oocytes and day 17 in vitro-produced and somatic cell nuclear transfer embryos. Biol Reprod.

[94] Davies W, Smith RJ, Kelsey G, Wilkinson LS (2004). Expression patterns of the novel imprinted genes Nap1l5 and Peg13 and their non-imprinted host genes in the adult mouse brain. Gene expression patterns : GEP.

[95] Smith RJ, Dean W, Konfortova G, Kelsey G (2003). Identification of novel imprinted genes in a genome-wide screen for maternal methylation. Genome Res.

[96] Schennink A, Stoop WM, Visker MH, Heck JM, Bovenhuis H, van der Poel JJ, van Valenberg HJ, van Arendonk JA (2007). DGAT1 underlies large genetic variation in milk-fat composition of dairy cows. Anim Genet.

[97] Khan MZ, Ma Y, Ma J, Xiao J, Liu Y, Liu S, Khan A, Khan IM, Cao Z (2021). Association of DGAT1 With Cattle, Buffalo, Goat, and Sheep Milk and Meat Production Traits. Front Vet Sci.

[98] Gutierrez-Gil B, Arranz JJ, Wiener P (2015). An interpretive review of selective sweep studies in Bos taurus cattle populations: identification of unique and shared selection signals across breeds. Front Genet.

[99] Nakatsu F, Messa M, Nandez R, Czapla H, Zou Y, Strittmatter SM, De Camilli P (2015). Sac2/INPP5F is an inositol 4-phosphatase that functions in the endocytic pathway. J Cell Biol.

[100] Choi JD, Underkoffler LA, Wood AJ, Collins JN, Williams PT, Golden JA, Schuster EF, Loomes KM, Oakey RJ (2005). A novel variant of Inpp5f is imprinted in brain, and its expression is correlated with differential methylation of an internal CpG island. Mol Cell Biol.

[101] Monk D, Arnaud P, Frost JM, Wood AJ, Cowley M, Martin-Trujillo A, Guillaumet-Adkins A, Iglesias Platas I, Camprubi C, Bourc'his D (2011). Human imprinted retrogenes exhibit non-canonical imprint chromatin signatures and reside in non-imprinted host genes. Nucleic Acids Res.

[102] Bird AP (1980). DNA methylation and the frequency of CpG in animal DNA. Nucleic Acids Res.

[103] Hock R, Furusawa T, Ueda T, Bustin M (2007). HMG chromosomal proteins in development and disease. Trends Cell Biol.

[104] Pryce JE, Hayes BJ, Bolormaa S, Goddard ME (2011). Polymorphic regions affecting human height also control stature in cattle. Genetics.

[105] Zhou X, Benson KF, Ashar HR, Chada K (1995). Mutation responsible for the mouse pygmy phenotype in the developmentally regulated factor HMGI-C. Nature.

[106] Carneiro M, Hu D, Archer J, Feng C, Afonso S, Chen C, Blanco-Aguiar JA, Garreau H, Boucher S, Ferreira PG et al: Dwarfism and Altered Craniofacial Development in Rabbits Is Caused by a 12.1 kb Deletion at the HMGA2 Locus. Genetics. 2017;205(2):955–965. 10.1534/genetics.116.196667 PMC5289862 27986804

[107] Makvandi-Nejad S, Hoffman GE, Allen JJ, Chu E, Gu E, Chandler AM, Loredo AI, Bellone RR, Mezey JG, Brooks SA (2012). Four loci explain 83% of size variation in the horse. PLoS ONE.

[108] Frischknecht M, Jagannathan V, Plattet P, Neuditschko M, Signer-Hasler H, Bachmann I, Pacholewska A, Drogemuller C, Dietschi E, Flury C (2015). A Non-Synonymous HMGA2 Variant Decreases Height in Shetland Ponies and Other Small Horses. PLoS ONE.

[109] Bouwman AC, Daetwyler HD, Chamberlain AJ, Ponce CH, Sargolzaei M, Schenkel FS, Sahana G, Govignon-Gion A, Boitard S, Dolezal M (2018). Meta-analysis of genome-wide association studies for cattle stature identifies common genes that regulate body size in mammals. Nat Genet.

[110] Mckittrick J, Chen P-Y, Bodde SG, Yang W, Novitskaya EE, Meyers MA (2012). The sructure, functions, and mechanical properties of keratin. JOM.

[111] Riemer J, Appenzeller-Herzog C, Johansson L, Bodenmiller B, Hartmann-Petersen R, Ellgaard L (2009). A luminal flavoprotein in endoplasmic reticulum-associated degradation. Proc Natl Acad Sci U S A.

[112] Liu R, Tearle R, Low WY, Chen T, Thomsen D, Smith TPL, Hiendleder S, Williams JL (2021). Distinctive gene expression patterns and imprinting signatures revealed in reciprocal crosses between cattle sub-species. BMC Genomics.

[113] Plassais J, Kim J, Davis BW, Karyadi DM, Hogan AN, Harris AC, Decker B, Parker HG, Ostrander EA (2019). Whole genome sequencing of canids reveals genomic regions under selection and variants influencing morphology. Nat Commun.

[114] Signer-Hasler H, Flury C, Haase B, Burger D, Simianer H, Leeb T, Rieder S (2012). A genome-wide association study reveals loci influencing height and other conformation traits in horses. PLoS ONE.

[115] Rubin CJ, Megens HJ, Martinez Barrio A, Maqbool K, Sayyab S, Schwochow D, Wang C, Carlborg O, Jern P, Jorgensen CB (2012). Strong signatures of selection in the domestic pig genome. Proc Natl Acad Sci U S A.

[116] Ruiz-Larranaga O, Langa J, Rendo F, Manzano C, Iriondo M, Estonba A (2018). Genomic selection signatures in sheep from the Western Pyrenees. Genet Sel Evol.

[117] Signer-Hasler H, Burren A, Ammann P, Drogemuller C, Flury C (2019). Runs of homozygosity and signatures of selection: a comparison among eight local Swiss sheep breeds. Anim Genet.

[118] Conway E, Jerman E, Healy E, Ito S, Holoch D, Oliviero G, Deevy O, Glancy E, Fitzpatrick DJ, Mucha M et al: A Family of Vertebrate-Specific Polycombs Encoded by the LCOR/LCORL Genes Balance PRC2 Subtype Activities. Mol Cell 2018, 70(3):408–421 e408. 10.1016/j.molcel.2018.03.005 29628311

[119] Margueron R, Reinberg D (2011). The Polycomb complex PRC2 and its mark in life. Nature.

[120] Wang Z, Hou X, Qu B, Wang J, Gao X, Li Q (2014). Pten regulates development and lactation in the mammary glands of dairy cows. PLoS ONE.

[121] Chen CC, Stairs DB, Boxer RB, Belka GK, Horseman ND, Alvarez JV, Chodosh LA (2012). Autocrine prolactin induced by the Pten-Akt pathway is required for lactation initiation and provides a direct link between the Akt and Stat5 pathways. Genes Dev.

[122] Weasner B, Salzer C, Kumar JP (2007). Sine oculis, a member of the SIX family of transcription factors, directs eye formation. Dev Biol.

[123] Nishiguchi S, Wood H, Kondoh H, Lovell-Badge R, Episkopou V (1998). Sox1 directly regulates the gamma-crystallin genes and is essential for lens development in mice. Genes Dev.

[124] Laclef C, Hamard G, Demignon J, Souil E, Houbron C, Maire P (2003). Altered myogenesis in Six1-deficient mice. Development.

[125] Ozaki H, Nakamura K, Funahashi J, Ikeda K, Yamada G, Tokano H, Okamura HO, Kitamura K, Muto S, Kotaki H (2004). Six1 controls patterning of the mouse otic vesicle. Development.

[126] Szczepny A, Hime GR, Loveland KL (2006). Expression of hedgehog signalling components in adult mouse testis. Developmental dynamics : an official publication of the American Association of Anatomists.

[127] Melo TP, Fortes MRS, Bresolin T, Mota LFM, Albuquerque LG, Carvalheiro R (2018). Multitrait meta-analysis identified genomic regions associated with sexual precocity in tropical beef cattle. J Anim Sci.

[128] Chandran U, Indu S, Kumar AT, Devi AN, Khan I, Srivastava D, Kumar PG (2016). Expression of Cnnm1 and Its Association with Stemness, Cell Cycle, and Differentiation in Spermatogenic Cells in Mouse Testis. Biol Reprod.

[129] Cacciola G, Chioccarelli T, Ricci G, Meccariello R, Fasano S, Pierantoni R, Cobellis G: The endocannabinoid system in vertebrate male reproduction: a comparative overview. Mol Cell Endocrinol 2008, 286(1–2 Suppl 1):S24–30. 10.1016/j.mce.2008.01.004 18342433

[130] Georges M, Charlier C, Hayes B (2019). Harnessing genomic information for livestock improvement. Nat Rev Genet.

[131] El Hajj N, Trapphoff T, Linke M, May A, Hansmann T, Kuhtz J, Reifenberg K, Heinzmann J, Niemann H, Daser A (2011). Limiting dilution bisulfite (pyro)sequencing reveals parent-specific methylation patterns in single early mouse embryos and bovine oocytes. Epigenetics.

[132] Wyss P, Song C, Bina M: Along the Bos Taurus genome, uncover candidate Imprinting Control Regions. bioRxiv 2021. 10.1186/s12864-022-08694-3 PMC9241299 35764919

[133] Li Z, Gilbert JA, Zhang Y, Zhang M, Qiu Q, Ramanujan K, Shavlakadze T, Eash JK, Scaramozza A, Goddeeris MM (2012). An HMGA2-IGF2BP2 axis regulates myoblast proliferation and myogenesis. Dev Cell.

[134] Kent WJ (2002). BLAT–the BLAST-like alignment tool. Genome Res.

[135] Zweig AS, Karolchik D, Kuhn RM, Haussler D, Kent WJ (2008). UCSC genome browser tutorial. Genomics.

[136] Lee CM, Barber GP, Casper J, Clawson H, Diekhans M, Gonzalez JN, Hinrichs AS, Lee BT, Nassar LR, Powell CC (2020). UCSC Genome Browser enters 20th year. Nucleic Acids Res.

[137] Bina M (2008). The genome browser at UCSC for locating genes, and much more!. Mol Biotechnol.

[138] Ziebarth JD, Bhattacharya A, Cui Y. CTCFBSDB 2.0: a database for CTCF-binding sites and genome organization. Nucleic Acids Res. 2013;41(Database issue):D188–194. 10.1093/nar/gks1165 PMC3531215 23193294

[139] Bina M: Assessment of the CTCF Binding Sites and Repeat-Positions Upstream the Human H19 Gene. bioRxiv 2018, https://www.biorxiv.org/content/10.1101/250407v1.

[140] ENCODE. A user's guide to the encyclopedia of DNA elements (ENCODE). PLoS Biol. 2011;9(4):e1001046. 10.1371/journal.pbio.1001046 PMC3079585 21526222

