# Supplementary material for: Along the Bos taurus genome, uncover candidate imprinting control regions
Source: BMC Genomics. 2022 Jun 28;23:478. doi: 10.1186/s12864-022-08694-3 (PMC9241299; doi:10.1186/s12864-022-08694-3)
Supplement: Supplementary file 1 — Additional file 1. [file 12864_2022_8694_MOESM1_ESM.pdf]

Additional file 1

GEO series GSE77444 offers a link to view results of chromatin immunoprecipitations (ChIPs) reported by Riso et al. [1]. The goal was to locate ICRs/gDMRs in chromatin prepared from mouse embryonic stem cells (ESCs) E14. Experimental type consisted of genome binding/occupancy profiling by high throughput sequencing [1]. From the extensive series, we selected to display datasets to locate ZFP57, KAP1, and H3K9me3 histone marks in chromatin. Reference [2] gives an overview of the functions of proteins that function in imprinted gene expression. Subsequent pages provide snapshots demonstrating that peaks in the density-plots occur precisely in regions associated with ZFP57, KAP1/TRIM28, and H3K9me3 marks in chromatin.

References

1. Riso V, Cammisa M, Kukreja H, Anvar Z, Verde G, Sparago A, Acurzio B, Lad S, Lonardo E, Sankar A *et al*: **ZFP57 maintains the parent-of-origin-specific expression of the imprinted genes and differentially affects non-imprinted targets in mouse embryonic stem cells**. *Nucleic Acids Res* 2016, **44**(17):8165-8178.
2. Strogantsev R, Ferguson-Smith AC: **Proteins involved in establishment and maintenance of imprinted methylation marks**. *Brief Funct Genomics* 2012, **11**(3):227-239.

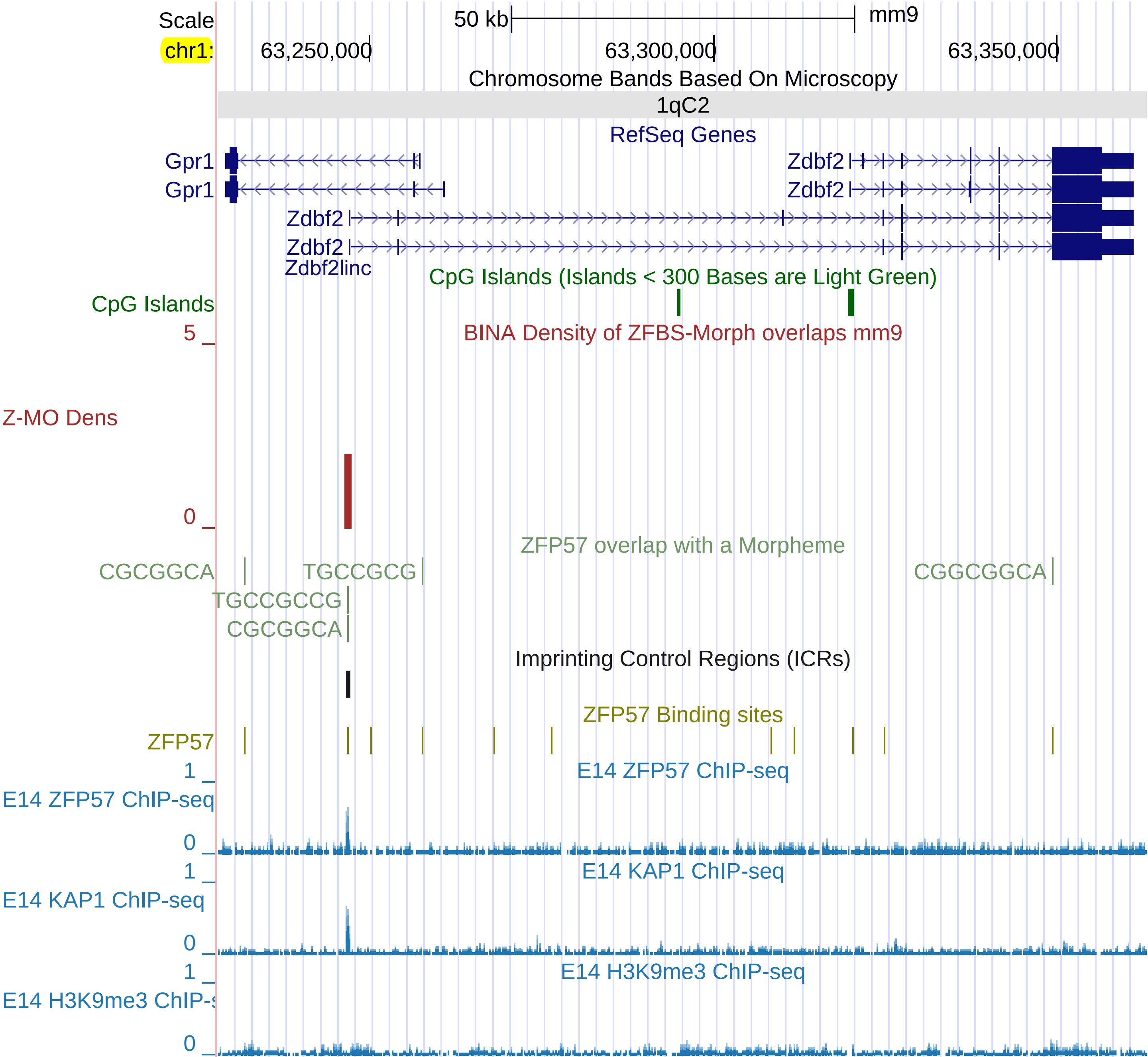
Scale
50 kb
mm9
chr1:
63,250,000
63,300,000
63,350,000
Chromosome Bands Based On Microscopy
1qC2
RefSeq Genes
Gpr1
Gpr1
Zdbf2
Zdbf2
Zdbf2
Zdbf2
Zdbf2linc
CpG Islands (Islands < 300 Bases are Light Green)
CpG Islands
5 _
BINA Density of ZFBS-Morph overlaps mm9
Z-MO Dens
0 _
ZFP57 overlap with a Morpheme
CGCGGCA
TGCCGCG
CGGCGGCA
TGCCGCCG
CGCGGCA
Imprinting Control Regions (ICRs)
ZFP57 Binding sites
ZFP57
1 _
E14 ZFP57 ChIP-seq
E14 ZFP57 ChIP-seq
0 _
1 _
E14 KAP1 ChIP-seq
E14 KAP1 ChIP-seq
0 _
1 _
E14 H3K9me3 ChIP-seq
E14 H3K9me3 ChIP-s
0 _

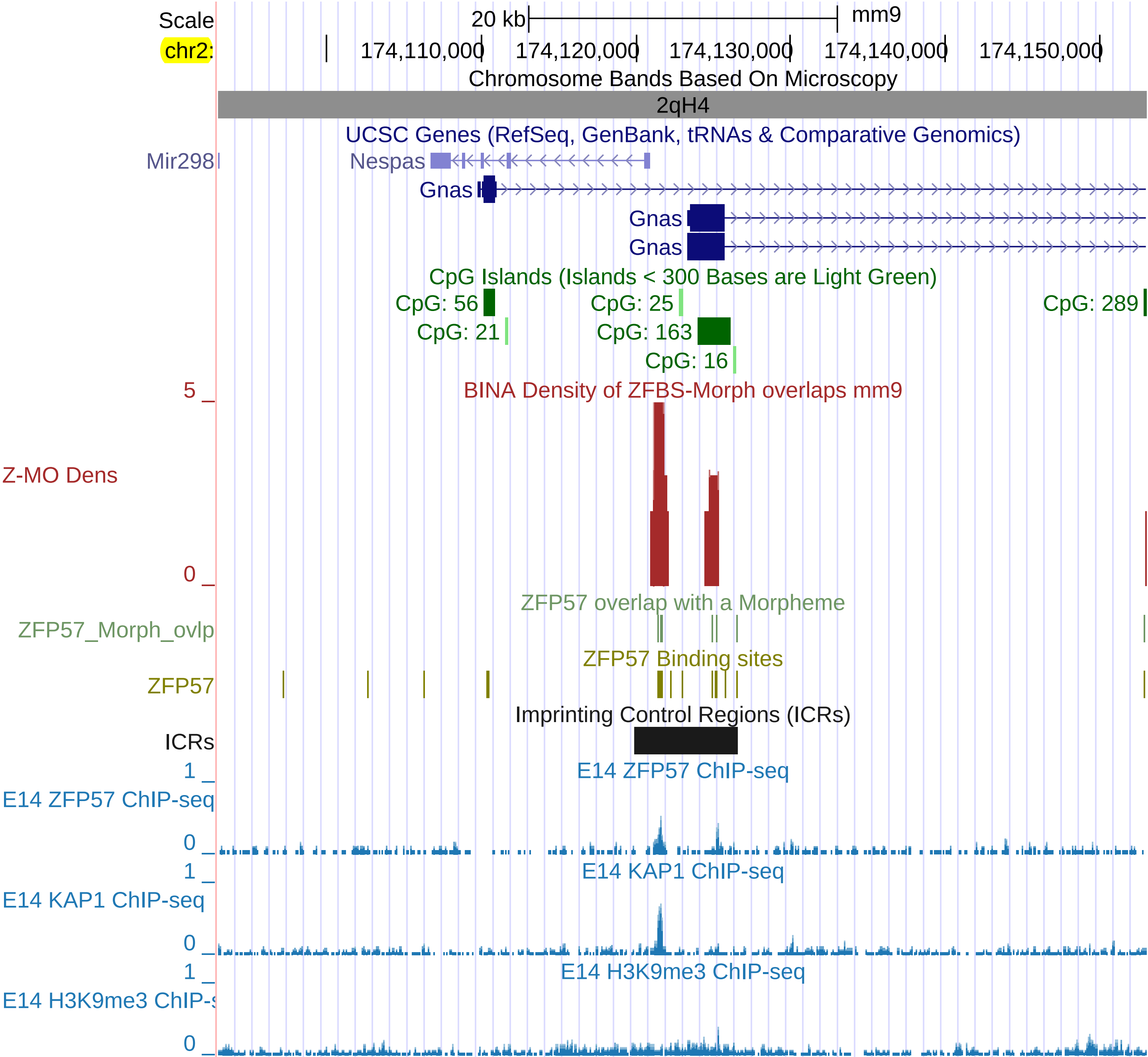
Scale
20 kb
mm9
chr2:
174,110,000
174,120,000
174,130,000
174,140,000
174,150,000
Chromosome Bands Based On Microscopy
2qH4
UCSC Genes (RefSeq, GenBank, tRNAs & Comparative Genomics)
Mir298
Nespas
Gnas
Gnas
Gnas
CpG Islands (Islands < 300 Bases are Light Green)
CpG: 56
CpG: 25
CpG: 289
CpG: 21
CpG: 163
CpG: 16
BINA Density of ZFBS-Morph overlaps mm9
5
Z-MO Dens
0
ZFP57 overlap with a Morpheme
ZFP57_Morph_ovlp
ZFP57 Binding sites
ZFP57
Imprinting Control Regions (ICRs)
ICRs
1
E14 ZFP57 ChIP-seq
E14 ZFP57 ChIP-seq
0
1
E14 KAP1 ChIP-seq
E14 KAP1 ChIP-seq
0
1
E14 H3K9me3 ChIP-seq
E14 H3K9me3 ChIP-s
0

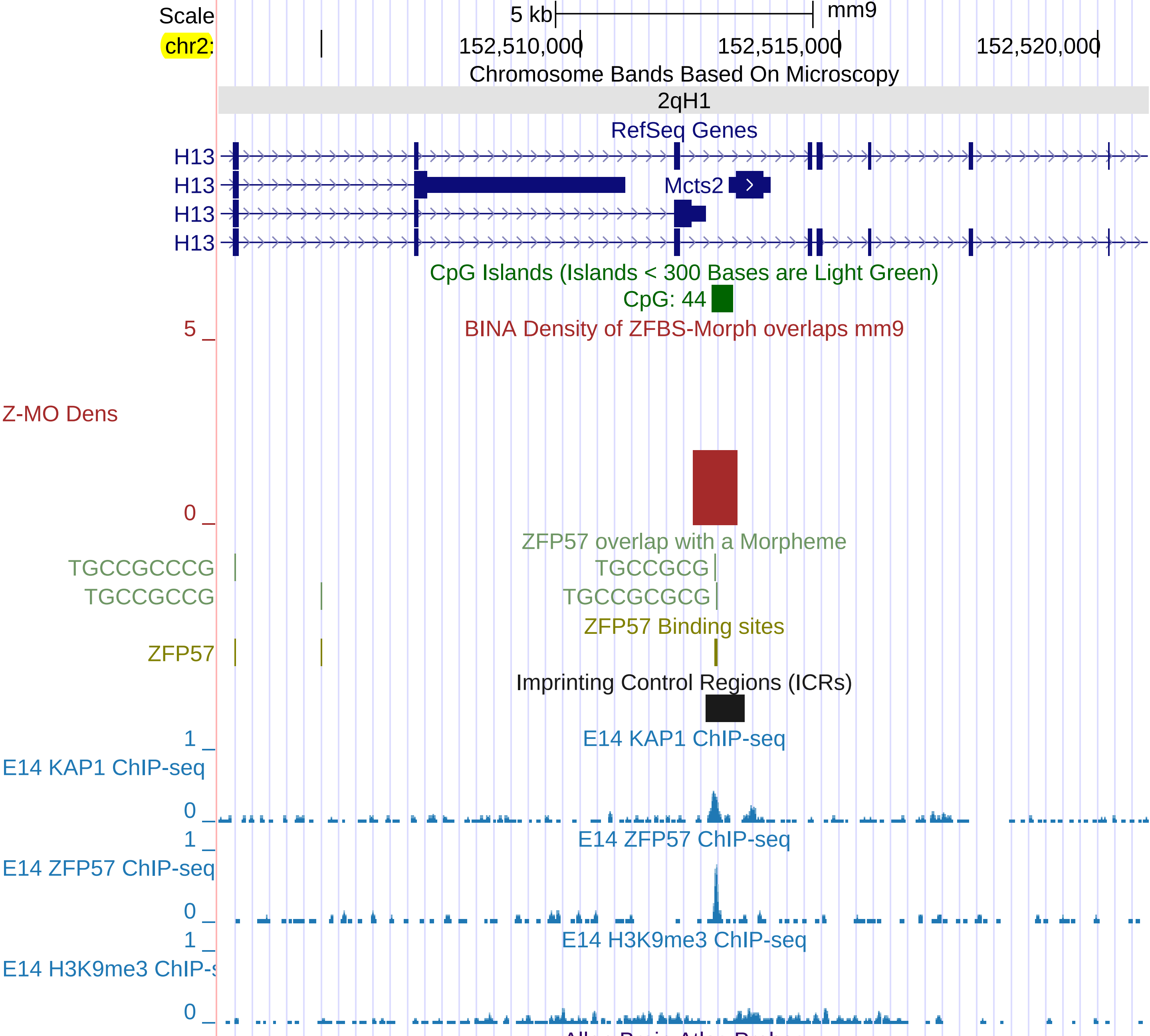
Scale
5 kb
mm9
chr2:
152,510,000
152,515,000
152,520,000
Chromosome Bands Based On Microscopy
2qH1
RefSeq Genes
H13
H13
Mcts2
H13
H13
CpG Islands (Islands < 300 Bases are Light Green)
CpG: 44
5 _
BINA Density of ZFBS-Morph overlaps mm9
Z-MO Dens
0 _
ZFP57 overlap with a Morpheme
TGCCGCCCG
TGCCGCG
TGCCGCCG
TGCCGCGCG
ZFP57 Binding sites
ZFP57
Imprinting Control Regions (ICRs)
1 _
E14 KAP1 ChIP-seq
E14 KAP1 ChIP-seq
0 _
1 _
E14 ZFP57 ChIP-seq
E14 ZFP57 ChIP-seq
0 _
1 _
E14 H3K9me3 ChIP-seq
E14 H3K9me3 ChIP-s
0 _

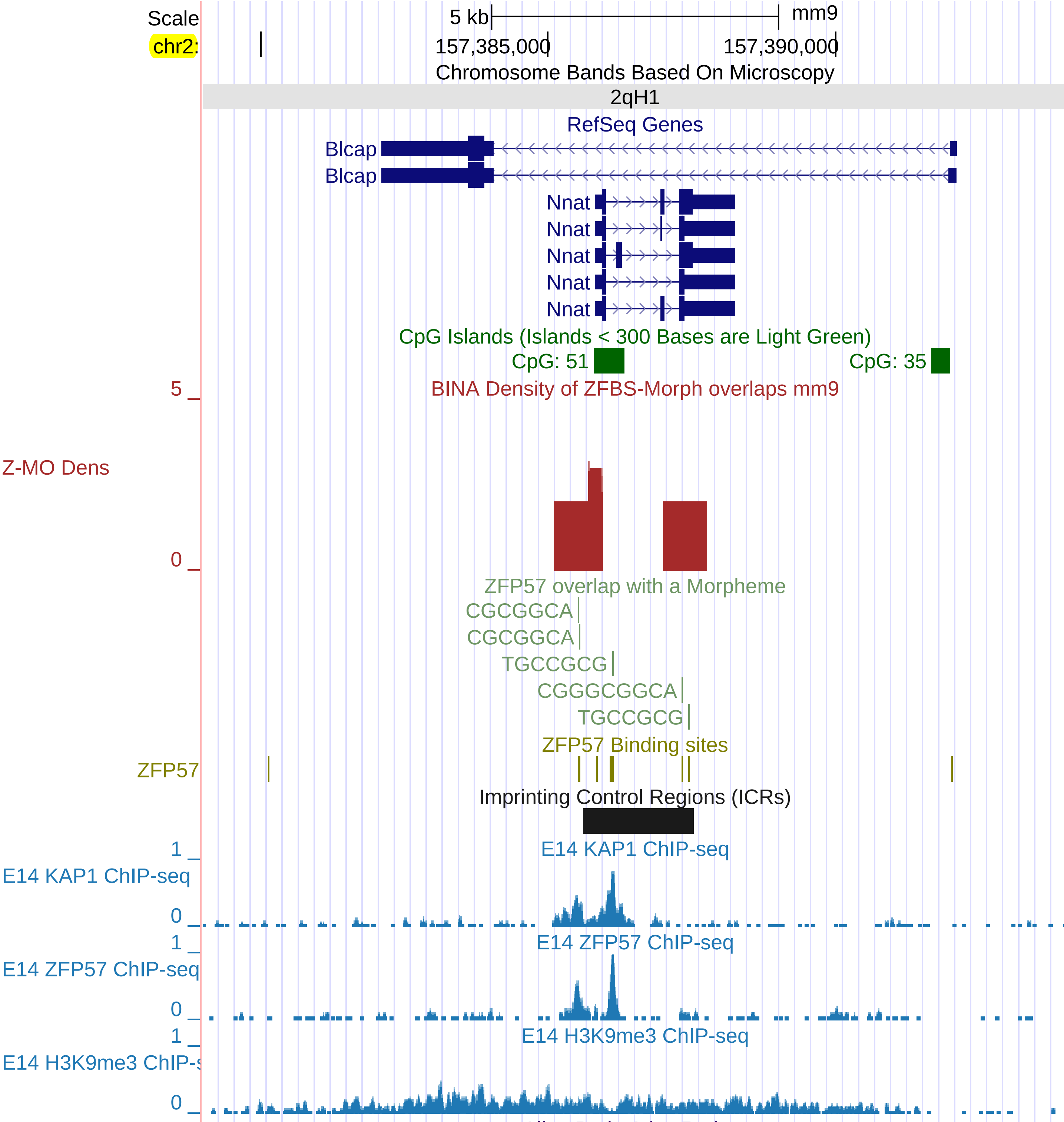
Scale
chr2:
5 kb
mm9
157,385,000
157,390,000
Chromosome Bands Based On Microscopy
2qH1
RefSeq Genes
Blcap
Blcap
Nnat
Nnat
Nnat
Nnat
Nnat
CpG Islands (Islands < 300 Bases are Light Green)
CpG: 51
CpG: 35
BINA Density of ZFBS-Morph overlaps mm9
5
Z-MO Dens
0
ZFP57 overlap with a Morpheme
CGCGGCA
CGCGGCA
TGCCGCG
CGGGCGGCA
TGCCGCG
ZFP57 Binding sites
ZFP57
Imprinting Control Regions (ICRs)
1
E14 KAP1 ChIP-seq
E14 KAP1 ChIP-seq
0
1
E14 ZFP57 ChIP-seq
E14 ZFP57 ChIP-seq
0
1
E14 H3K9me3 ChIP-seq
0

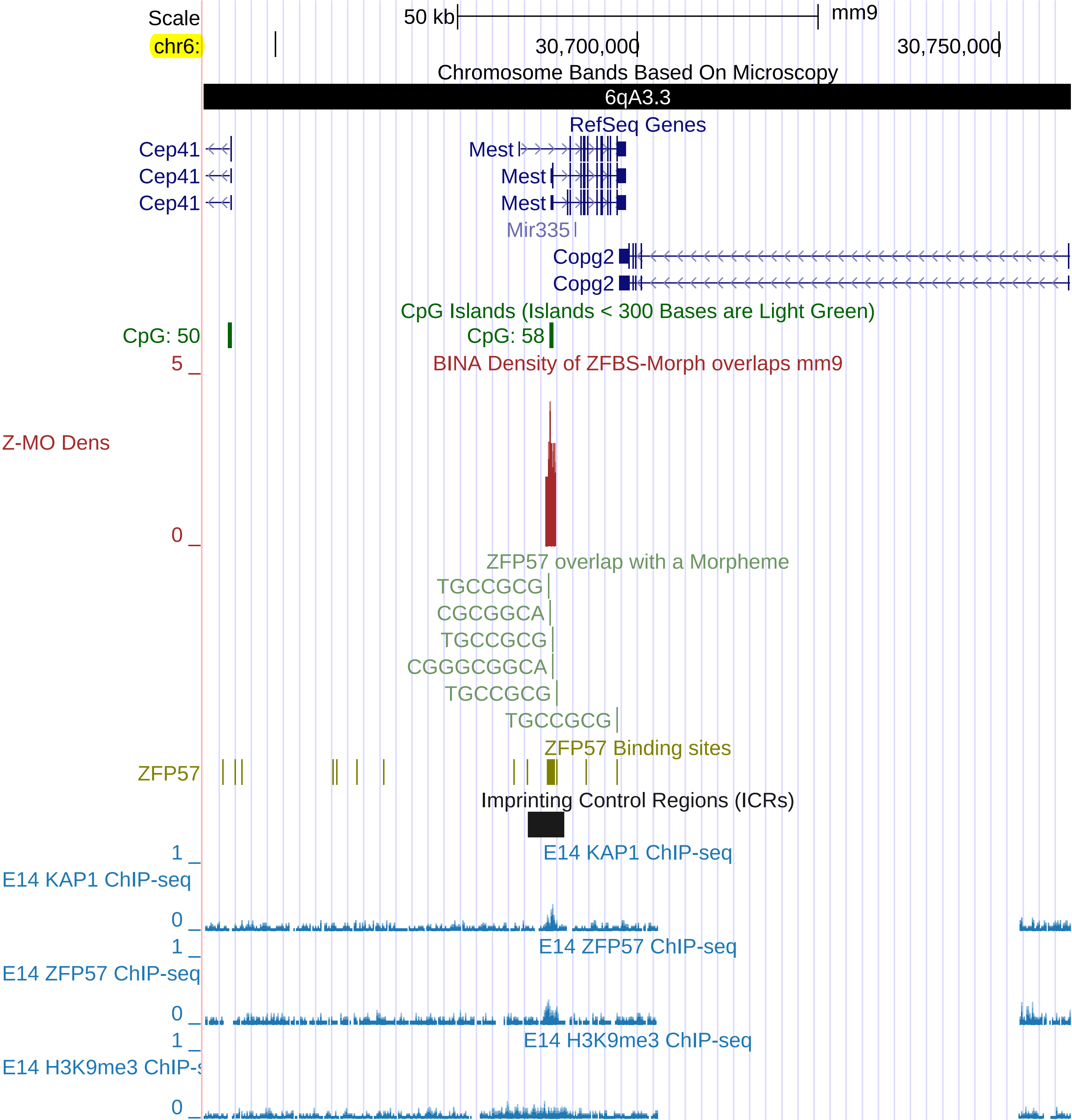
Scale
50 kb
mm9
chr6:
30,700,000
30,750,000
Chromosome Bands Based On Microscopy
6qA3.3
RefSeq Genes
Cep41
Cep41
Cep41
Mest
Mest
Mest
Mir335
Copg2
Copg2
CpG Islands (Islands < 300 Bases are Light Green)
CpG: 50
CpG: 58
5 _
BINA Density of ZFBS-Morph overlaps mm9
Z-MO Dens
0 _
ZFP57 overlap with a Morpheme
TGCCGCG
CGCGGCA
TGCCGCG
CGGGCGGCA
TGCCGCG
TGCCGCG
ZFP57 Binding sites
ZFP57
Imprinting Control Regions (ICRs)
1 _
E14 KAP1 ChIP-seq
E14 KAP1 ChIP-seq
0 _
1 _
E14 ZFP57 ChIP-seq
E14 ZFP57 ChIP-seq
0 _
1 _
E14 H3K9me3 ChIP-seq
0 _

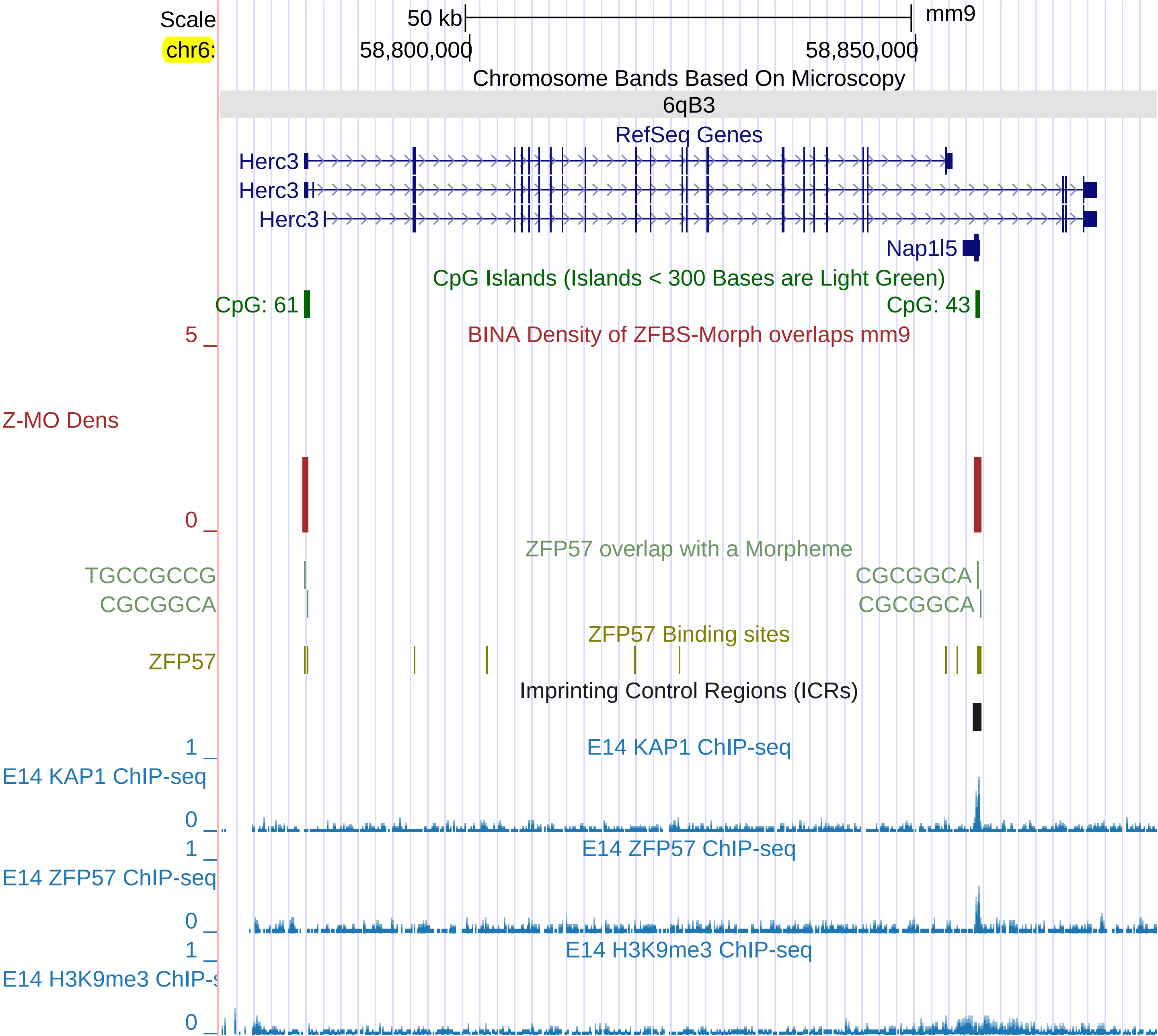
Scale
50 kb
mm9
chr6:
58,800,000
58,850,000
Chromosome Bands Based On Microscopy
6qB3
RefSeq Genes
Herc3
Herc3
Herc3
Nap1l5
CpG Islands (Islands < 300 Bases are Light Green)
CpG: 61
CpG: 43
5 _
BINA Density of ZFBS-Morph overlaps mm9
Z-MO Dens
0 _
ZFP57 overlap with a Morpheme
TGCCGCCG
CGCGGCA
CGCGGCA
CGCGGCA
ZFP57 Binding sites
ZFP57
Imprinting Control Regions (ICRs)
1 _
E14 KAP1 ChIP-seq
E14 KAP1 ChIP-seq
0 _
1 _
E14 ZFP57 ChIP-seq
E14 ZFP57 ChIP-seq
0 _
1 _
E14 H3K9me3 ChIP-seq
E14 H3K9me3 ChIP-s
0 _

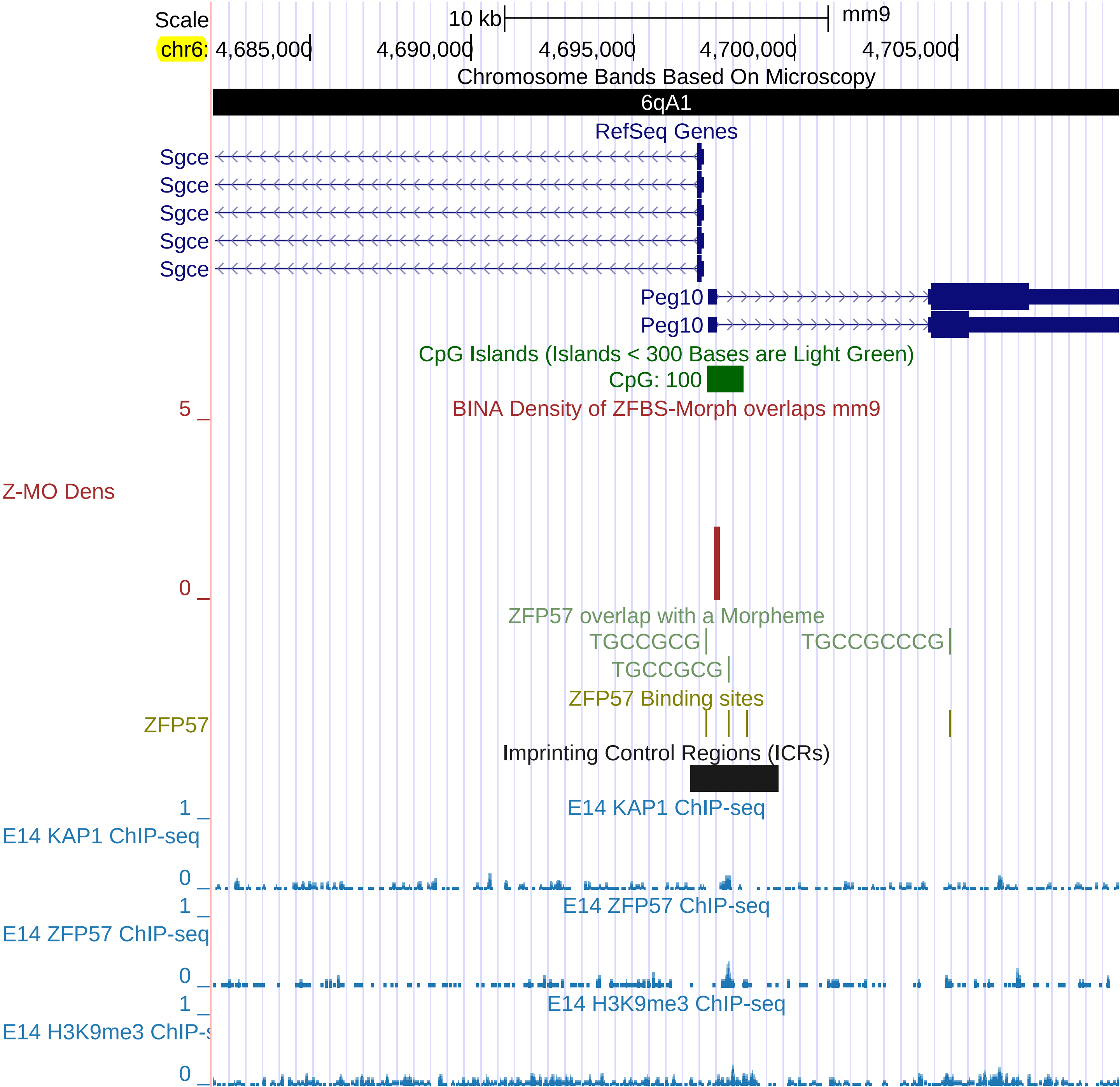
Scale
10 kb
mm9
chr6:
4,685,000
4,690,000
4,695,000
4,700,000
4,705,000
Chromosome Bands Based On Microscopy
6qA1
RefSeq Genes
Sgce
Sgce
Sgce
Sgce
Sgce
Peg10
Peg10
CpG Islands (Islands < 300 Bases are Light Green)
CpG: 100
BINA Density of ZFBS-Morph overlaps mm9
5 _
Z-MO Dens
0 _
ZFP57 overlap with a Morpheme
TGCCGCG
TGCCGCCCG
TGCCGCG
ZFP57 Binding sites
ZFP57
Imprinting Control Regions (ICRs)
1 _
E14 KAP1 ChIP-seq
E14 KAP1 ChIP-seq
0 _
1 _
E14 ZFP57 ChIP-seq
E14 ZFP57 ChIP-seq
0 _
1 _
E14 H3K9me3 ChIP-seq
0 _

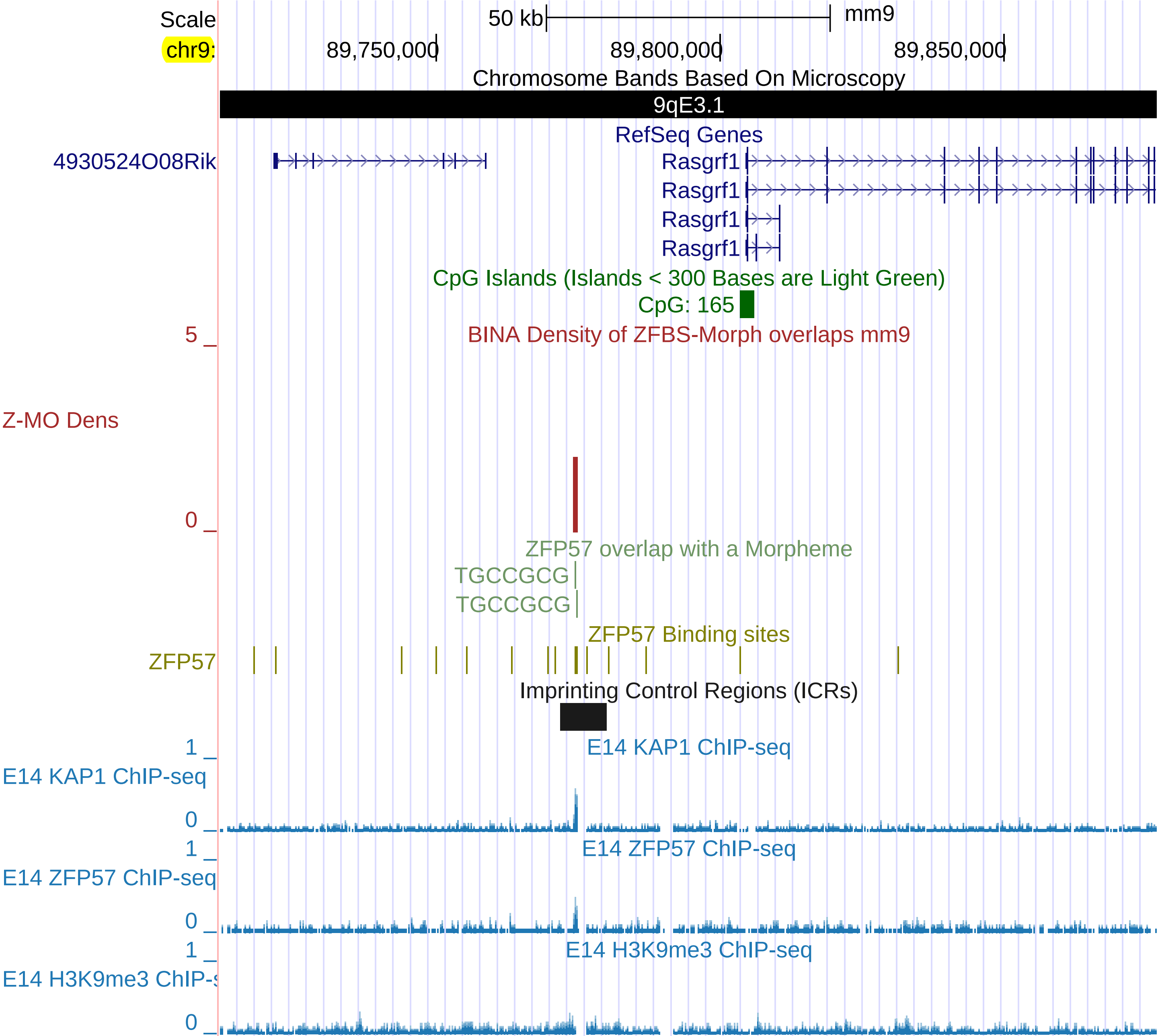
Scale
50 kb
mm9
chr9:
89,750,000
89,800,000
89,850,000
Chromosome Bands Based On Microscopy
9qE3.1
RefSeq Genes
4930524O08Rik
Rasgrf1
Rasgrf1
Rasgrf1
Rasgrf1
CpG Islands (Islands < 300 Bases are Light Green)
CpG: 165
5
BINA Density of ZFBS-Morph overlaps mm9
Z-MO Dens
0
ZFP57 overlap with a Morpheme
TGCCGCG
TGCCGCG
ZFP57 Binding sites
ZFP57
Imprinting Control Regions (ICRs)
1
E14 KAP1 ChIP-seq
E14 KAP1 ChIP-seq
0
1
E14 ZFP57 ChIP-seq
E14 ZFP57 ChIP-seq
0
1
E14 H3K9me3 ChIP-seq
E14 H3K9me3 ChIP-s
0

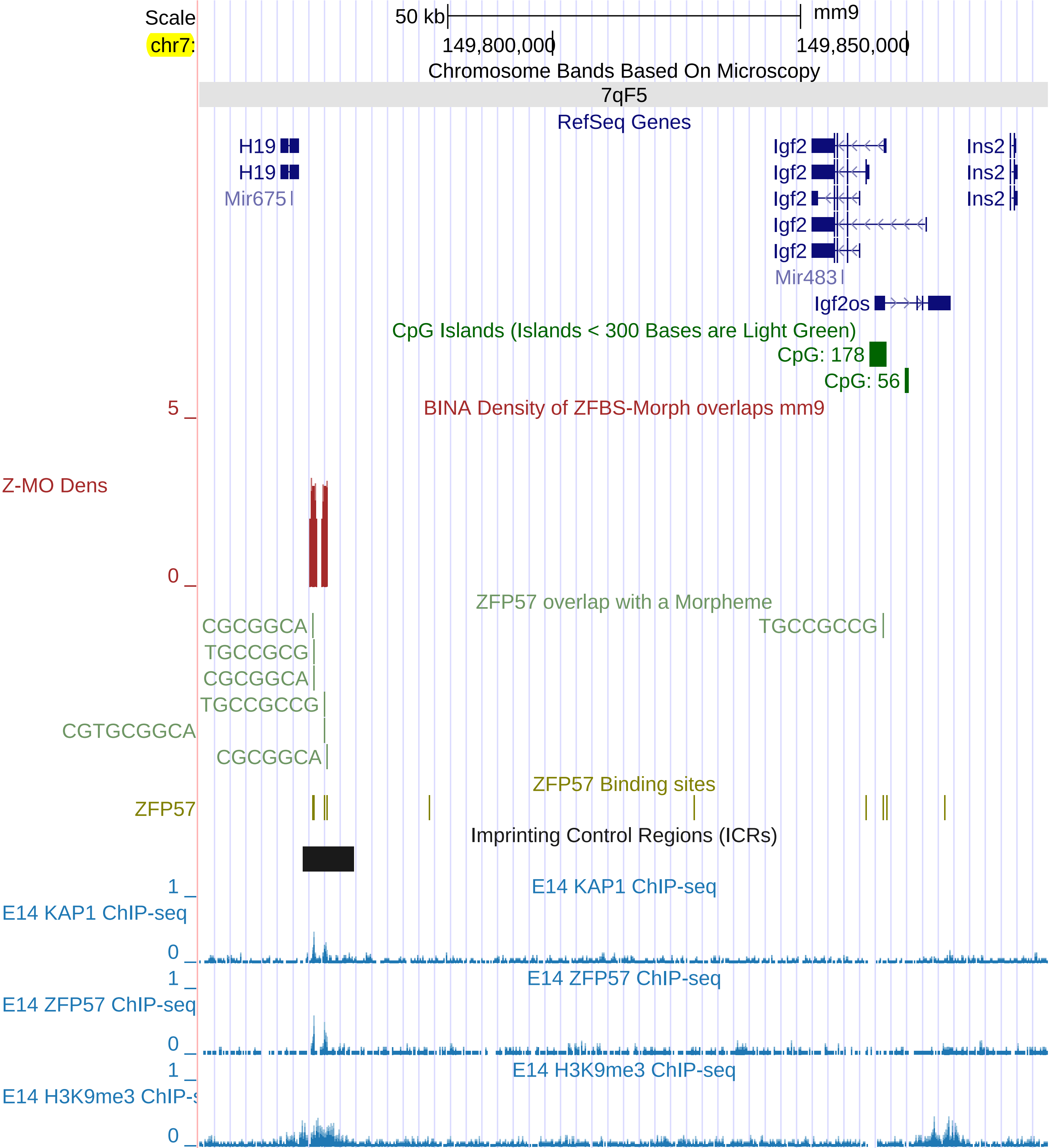
Scale
chr7:
50 kb
mm9
149,800,000
149,850,000
Chromosome Bands Based On Microscopy
7qF5
RefSeq Genes
H19
H19
Mir675
Igf2
Igf2
Igf2
Igf2
Igf2
Mir483
Igf2os
Ins2
Ins2
Ins2
CpG Islands (Islands < 300 Bases are Light Green)
CpG: 178
CpG: 56
5 _
BINA Density of ZFBS-Morph overlaps mm9
Z-MO Dens
0 _
ZFP57 overlap with a Morpheme
CGCGGCA
TGCCGCG
CGCGGCA
TGCCGCCG
CGTGCGGCA
CGCGGCA
TGCCGCCG
ZFP57 Binding sites
ZFP57
Imprinting Control Regions (ICRs)
1 _
E14 KAP1 ChIP-seq
E14 KAP1 ChIP-seq
0 _
1 _
E14 ZFP57 ChIP-seq
E14 ZFP57 ChIP-seq
0 _
1 _
E14 H3K9me3 ChIP-seq
E14 H3K9me3 ChIP-s
0 _

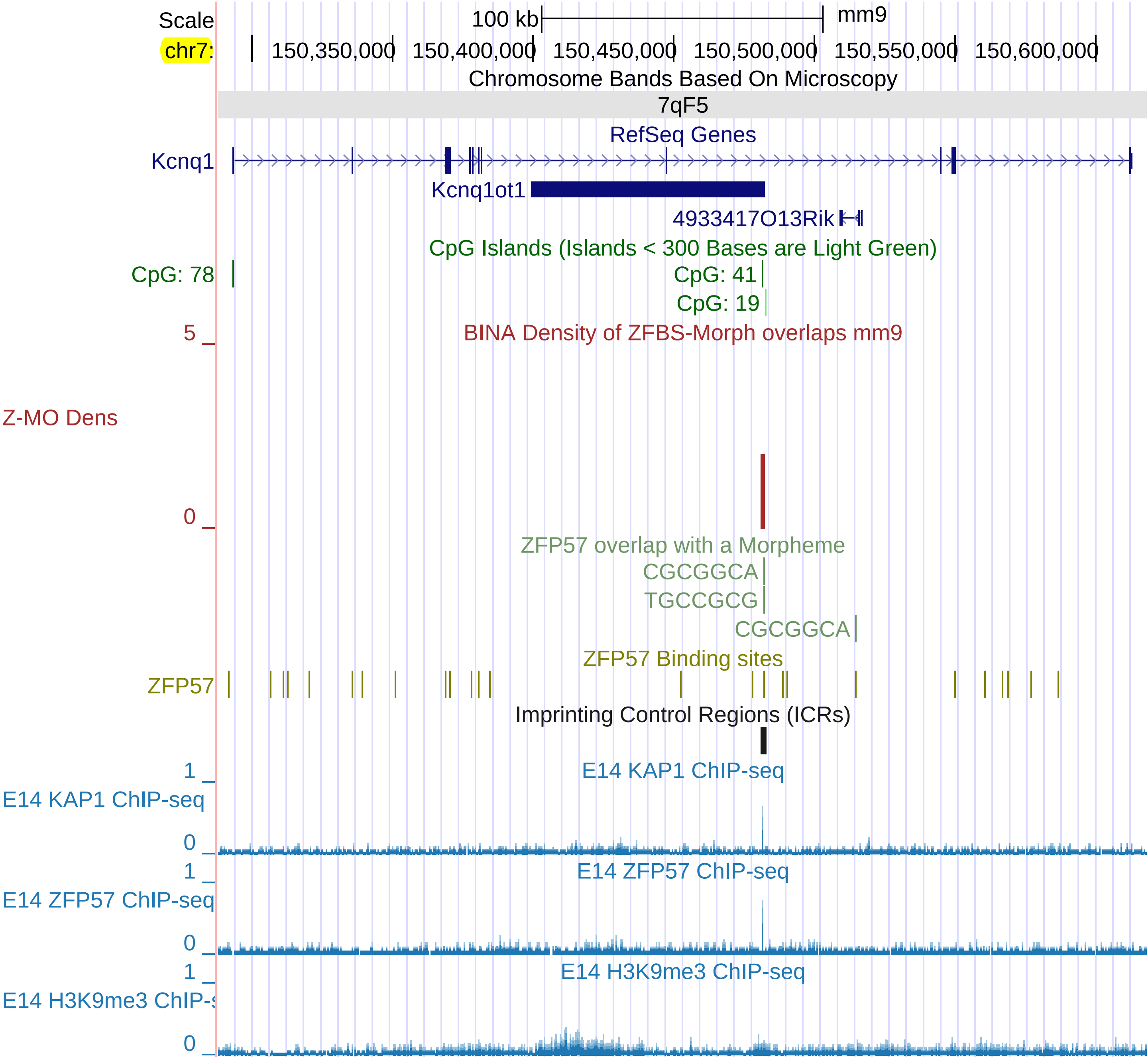
Scale
100 kb
mm9
chr7:
150,350,000
150,400,000
150,450,000
150,500,000
150,550,000
150,600,000
Chromosome Bands Based On Microscopy
7qF5
RefSeq Genes
Kcnq1
Kcnq1ot1
4933417O13Rik
CpG Islands (Islands < 300 Bases are Light Green)
CpG: 78
CpG: 41
CpG: 19
BINA Density of ZFBS-Morph overlaps mm9
5
Z-MO Dens
0
ZFP57 overlap with a Morpheme
CGCGGCA
TGCCGCG
CGCGGCA
ZFP57 Binding sites
ZFP57
Imprinting Control Regions (ICRs)
1
E14 KAP1 ChIP-seq
E14 KAP1 ChIP-seq
0
1
E14 ZFP57 ChIP-seq
E14 ZFP57 ChIP-seq
0
1
E14 H3K9me3 ChIP-seq
0

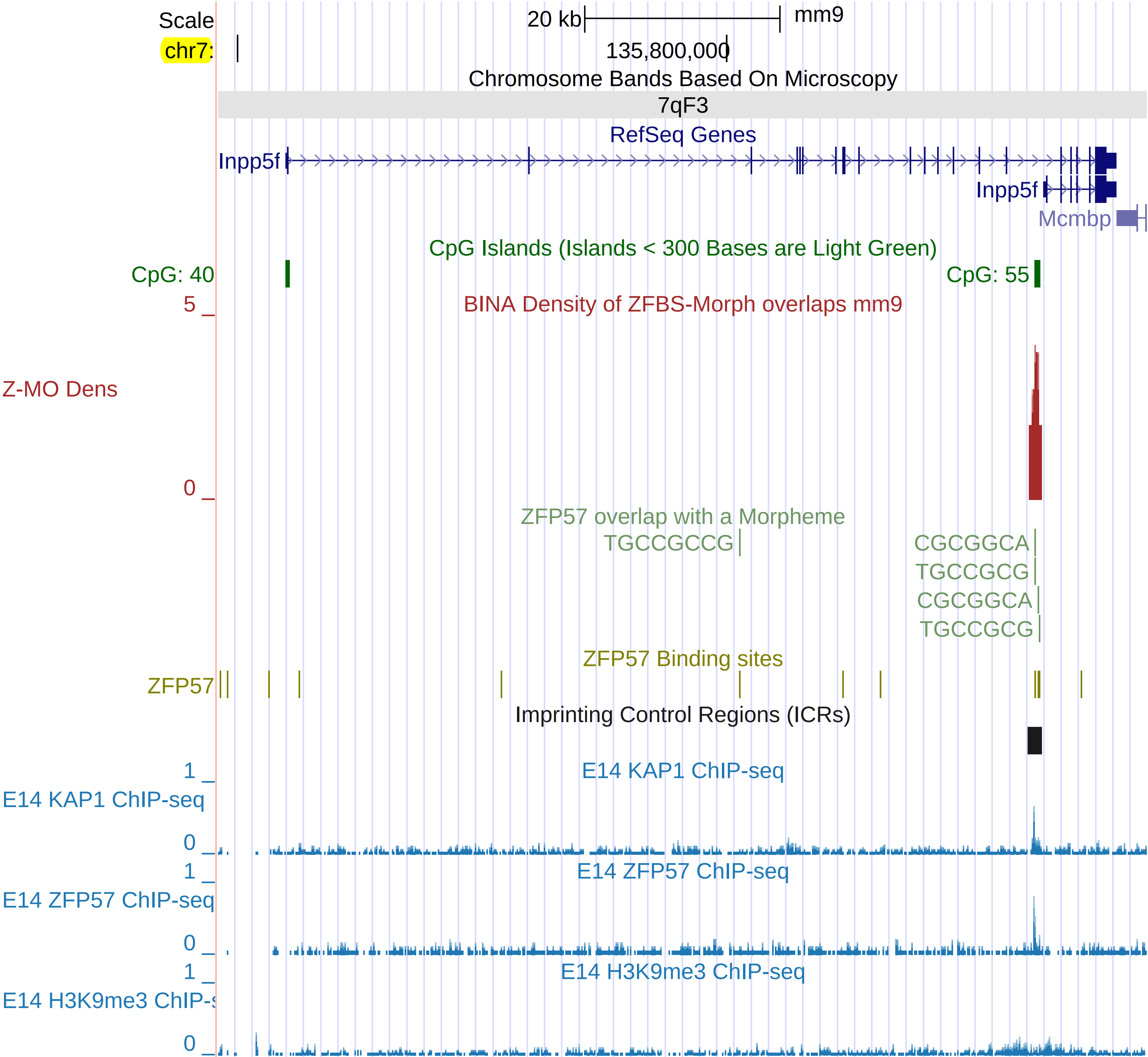
Scale
20 kb
mm9
chr7:
135,800,000
Chromosome Bands Based On Microscopy
7qF3
RefSeq Genes
Inpp5f
Inpp5f
Mcmbp
CpG Islands (Islands < 300 Bases are Light Green)
CpG: 40
CpG: 55
5 _
BINA Density of ZFBS-Morph overlaps mm9
Z-MO Dens
0 _
ZFP57 overlap with a Morpheme
TGCCGCCG
CGCGGCA
TGCCGCG
CGCGGCA
TGCCGCG
ZFP57 Binding sites
ZFP57
Imprinting Control Regions (ICRs)
1 _
E14 KAP1 ChIP-seq
E14 KAP1 ChIP-seq
0 _
1 _
E14 ZFP57 ChIP-seq
E14 ZFP57 ChIP-seq
0 _
1 _
E14 H3K9me3 ChIP-seq
E14 H3K9me3 ChIP-s
0 _

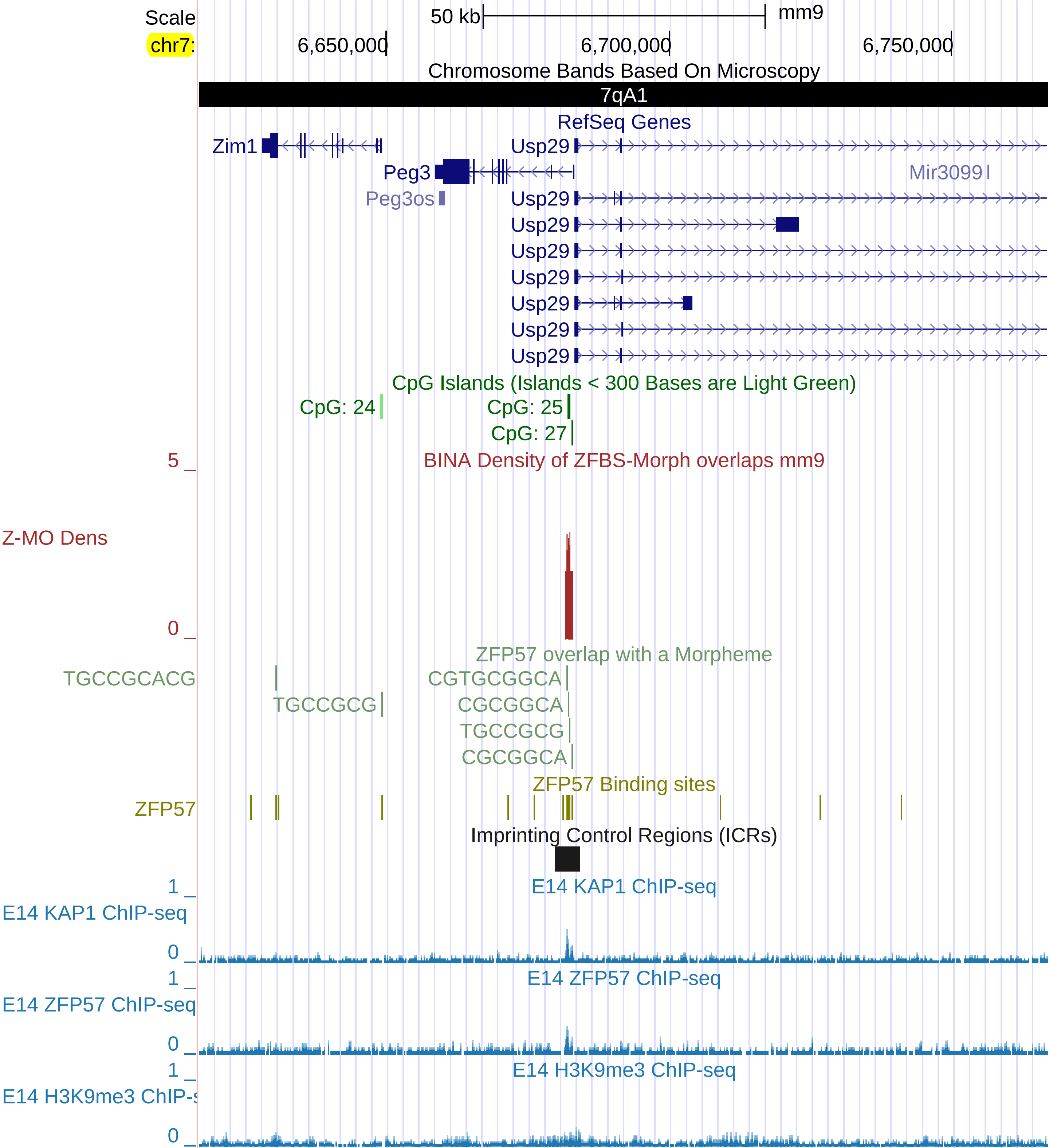
Scale
50 kb
mm9
chr7:
6,650,000
6,700,000
6,750,000
Chromosome Bands Based On Microscopy
7qA1
RefSeq Genes
Zim1
Usp29
Peg3
Mir3099
Peg3os
CpG Islands (Islands < 300 Bases are Light Green)
CpG: 24
CpG: 25
CpG: 27
BINA Density of ZFBS-Morph overlaps mm9
Z-MO Dens
5
0
ZFP57 overlap with a Morpheme
TGCCGCACG
CGTGCGGCA
TGCCGCG
CGCGGCA
TGCCGCG
CGCGGCA
ZFP57 Binding sites
ZFP57
Imprinting Control Regions (ICRs)
E14 KAP1 ChIP-seq
E14 ZFP57 ChIP-seq
E14 H3K9me3 ChIP-seq
1
0

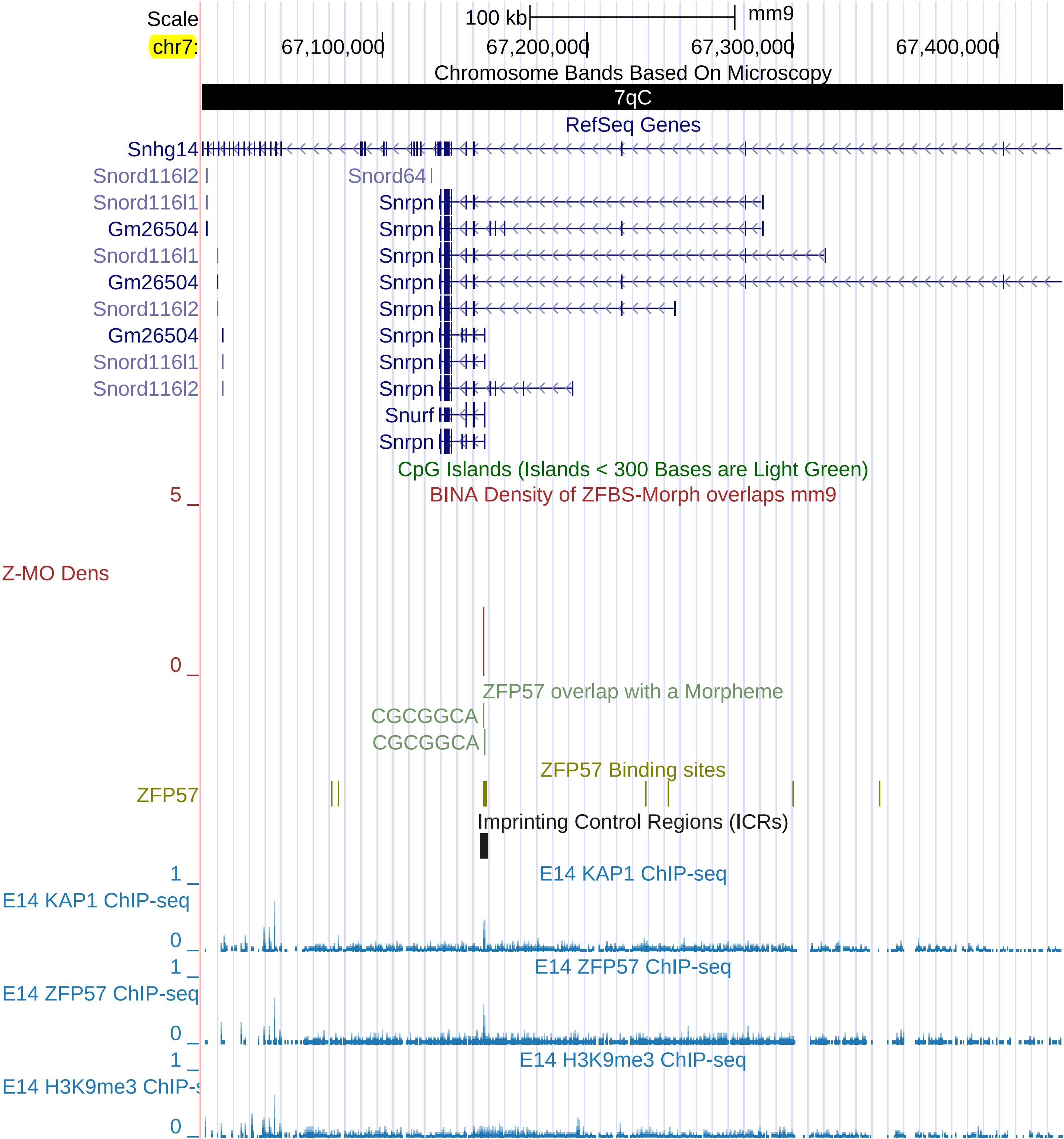
Scale
chr7:
100 kb
mm9
67,100,000
67,200,000
67,300,000
67,400,000
Chromosome Bands Based On Microscopy
7qC
RefSeq Genes
Snhg14
Snord116l2
Snord64
Snord116l1
Snrpn
Gm26504
Snord116l1
Gm26504
Snord116l2
Gm26504
Snord116l1
Snord116l2
Snurf
CpG Islands (Islands < 300 Bases are Light Green)
BINA Density of ZFBS-Morph overlaps mm9
5
Z-MO Dens
0
ZFP57 overlap with a Morpheme
CGCGGCA
CGCGGCA
ZFP57 Binding sites
ZFP57
Imprinting Control Regions (ICRs)
E14 KAP1 ChIP-seq
1
0
E14 ZFP57 ChIP-seq
1
0
E14 H3K9me3 ChIP-seq
1
0

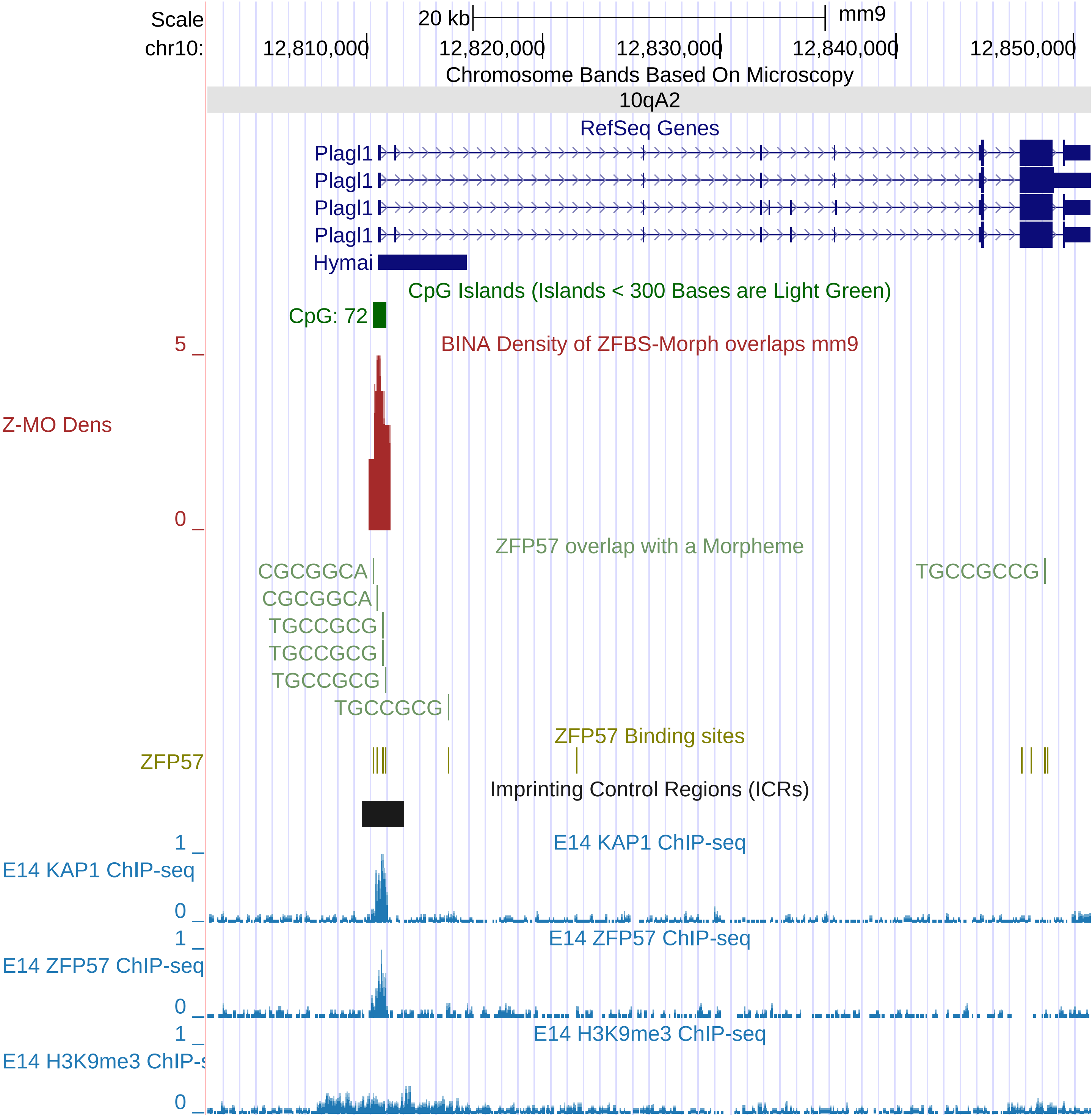

Scale
20 kb
mm9
chr10:
12,810,000
12,820,000
12,830,000
12,840,000
12,850,000
Chromosome Bands Based On Microscopy
10qA2
RefSeq Genes
Plagl1
Plagl1
Plagl1
Plagl1
Hymai
CpG Islands (Islands < 300 Bases are Light Green)
CpG: 72
5 _
BINA Density of ZFBS-Morph overlaps mm9
Z-MO Dens
0 _
ZFP57 overlap with a Morpheme
CGCGGCA
CGCGGCA
TGCCGCG
TGCCGCG
TGCCGCG
TGCCGCG
TGCCGCCG
ZFP57 Binding sites
ZFP57
Imprinting Control Regions (ICRs)
1 _
E14 KAP1 ChIP-seq
E14 KAP1 ChIP-seq
0 _
1 _
E14 ZFP57 ChIP-seq
E14 ZFP57 ChIP-seq
0 _
1 _
E14 H3K9me3 ChIP-seq
0 _

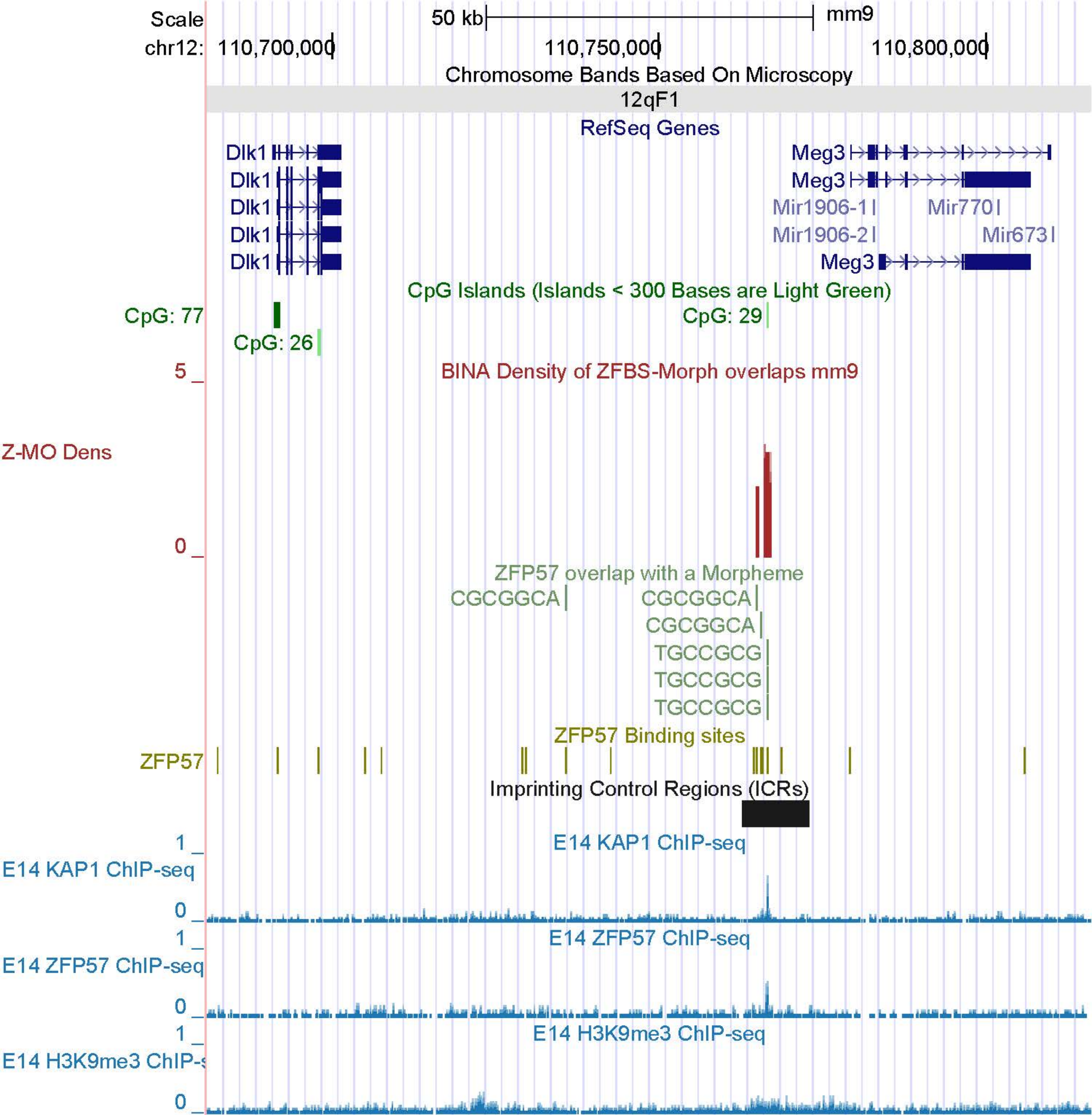
Scale
50 kb
mm9
chr12: 110,700,000
110,750,000
110,800,000
Chromosome Bands Based On Microscopy
12qF1
RefSeq Genes
Dlk1
Dlk1
Dlk1
Dlk1
Dlk1
Meg3
Meg3
Mir1906-1
Mir770
Mir1906-2
Mir673
Meg3
CpG Islands (Islands < 300 Bases are Light Green)
CpG: 77
CpG: 29
CpG: 26
5
BINA Density of ZFBS-Morph overlaps mm9
Z-MO Dens
0
ZFP57 overlap with a Morpheme
CGCGGCA
CGCGGCA
CGCGGCA
TGCCGCG
TGCCGCG
TGCCGCG
ZFP57 Binding sites
ZFP57
Imprinting Control Regions (ICRs)
1
E14 KAP1 ChIP-seq
E14 KAP1 ChIP-seq
0
1
E14 ZFP57 ChIP-seq
E14 ZFP57 ChIP-seq
0
1
E14 H3K9me3 ChIP-seq
E14 H3K9me3 ChIP-s
0

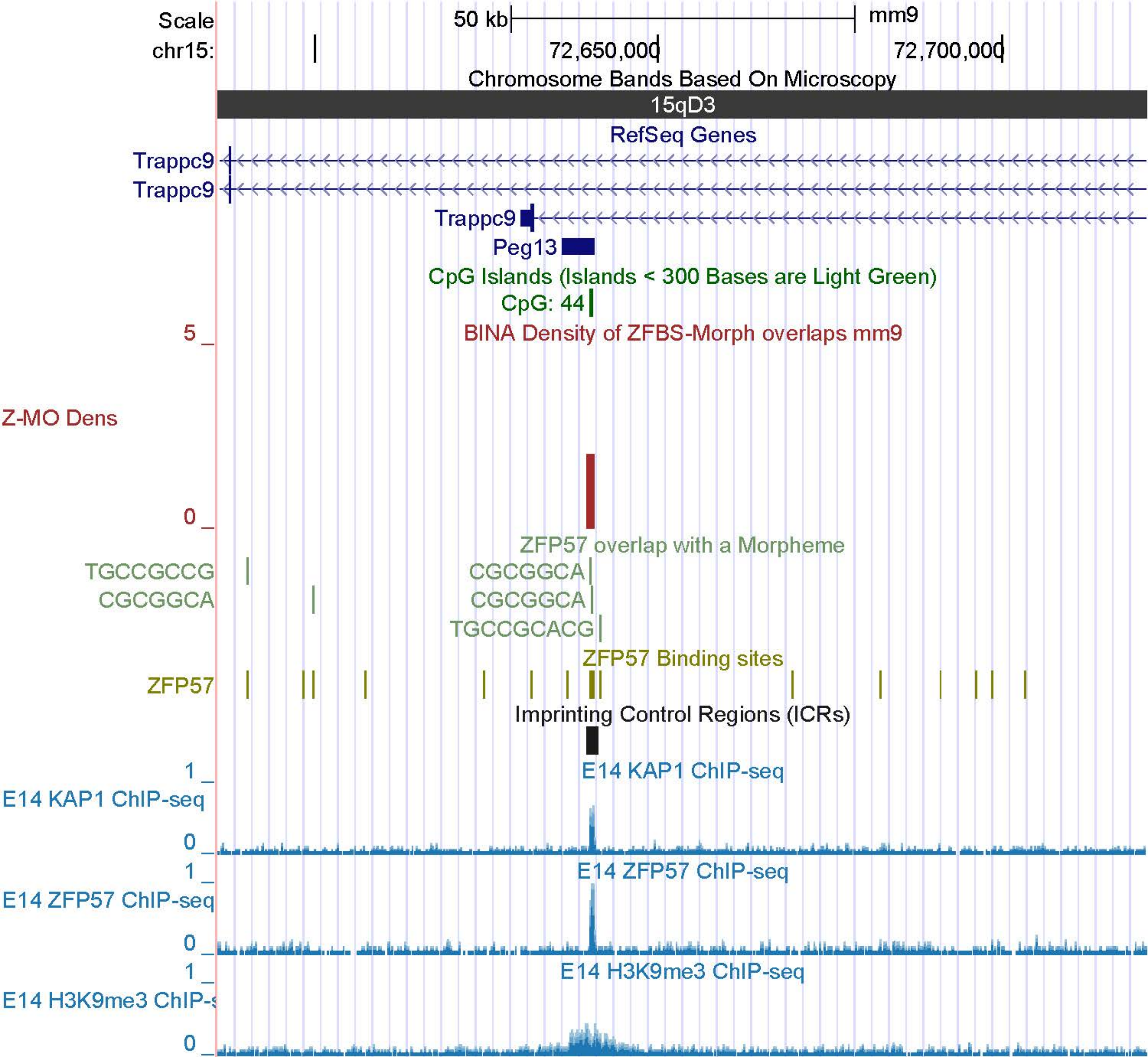

Scale
50 kb
mm9
chr15:
72,650,000
72,700,000
Chromosome Bands Based On Microscopy
15qD3
RefSeq Genes
Trappc9
Trappc9
Trappc9
Peg13
CpG Islands (Islands < 300 Bases are Light Green)
CpG: 44
5 _
BINA Density of ZFBS-Morph overlaps mm9
Z-MO Dens
0 _
ZFP57 overlap with a Morpheme
TGCCGCCG
CGCGGCA
CGCGGCA
CGCGGCA
TGCCGCACG
ZFP57 Binding sites
ZFP57
Imprinting Control Regions (ICRs)
1 _
E14 KAP1 ChIP-seq
E14 KAP1 ChIP-seq
0 _
1 _
E14 ZFP57 ChIP-seq
E14 ZFP57 ChIP-seq
0 _
1 _
E14 H3K9me3 ChIP-seq
E14 H3K9me3 ChIP-s
0 _

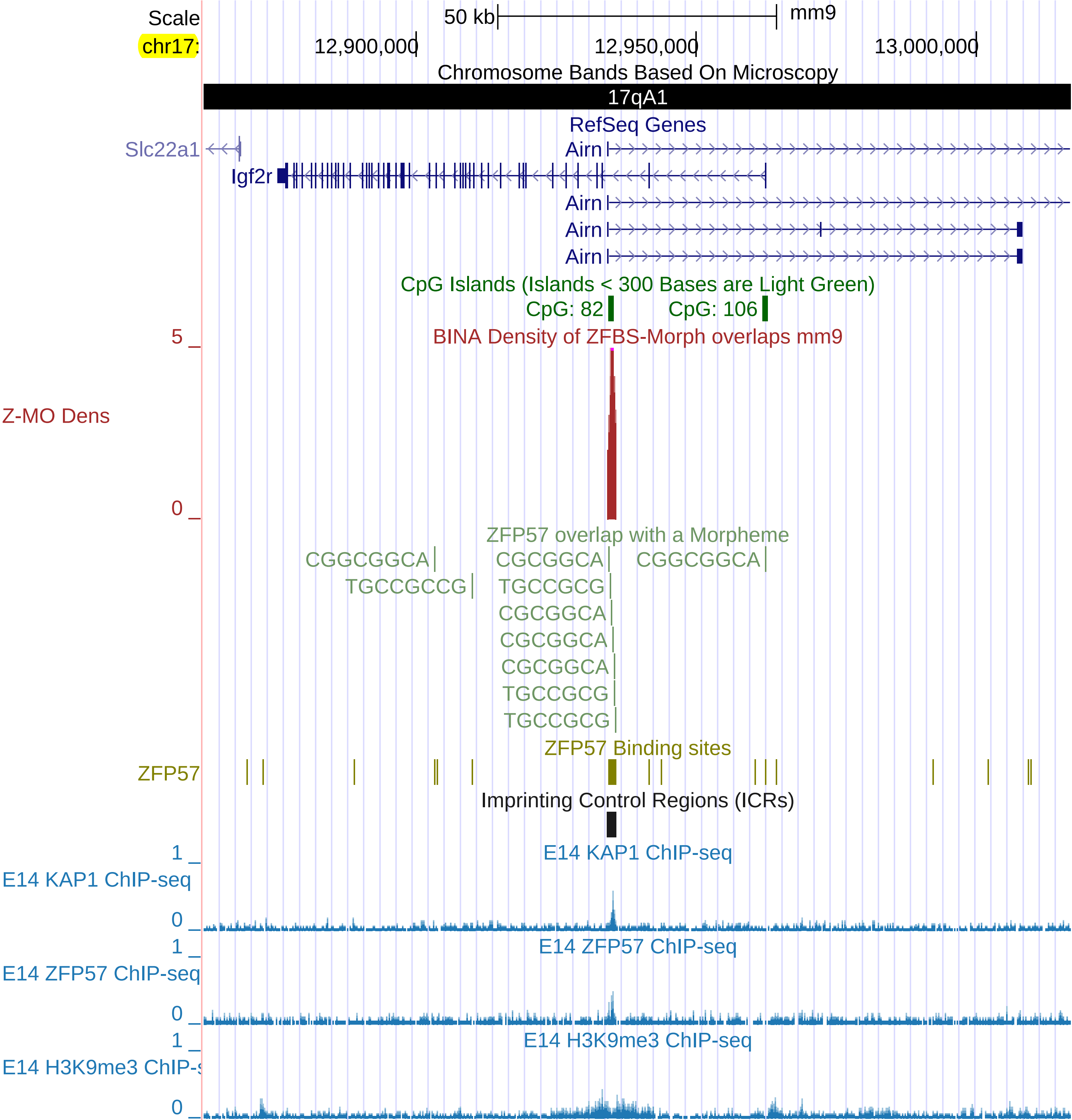

Scale
50 kb
mm9
chr17:
12,900,000
12,950,000
13,000,000
Chromosome Bands Based On Microscopy
17qA1
RefSeq Genes
Slc22a1
Igf2r
Airn
Airn
Airn
Airn
CpG Islands (Islands < 300 Bases are Light Green)
CpG: 82
CpG: 106
5
Z-MO Dens
0
BINA Density of ZFBS-Morph overlaps mm9
ZFP57 overlap with a Morpheme
CGGCGGCA
CGCGGCA
CGGCGGCA
TGCCGCCG
TGCCGCG
CGCGGCA
CGCGGCA
CGCGGCA
TGCCGCG
TGCCGCG
ZFP57 Binding sites
ZFP57
Imprinting Control Regions (ICRs)
1
E14 KAP1 ChIP-seq
0
E14 KAP1 ChIP-seq
1
E14 ZFP57 ChIP-seq
0
E14 ZFP57 ChIP-seq
1
E14 H3K9me3 ChIP-seq
0
