# Supplementary material for: Along the Bos taurus genome, uncover candidate imprinting control regions
Source: BMC Genomics. 2022 Jun 28;23:478. doi: 10.1186/s12864-022-08694-3 (PMC9241299; doi:10.1186/s12864-022-08694-3)
Supplement: Supplementary file 2 — Additional file 2. [file 12864_2022_8694_MOESM2_ESM.pdf]

Supplemental Figures

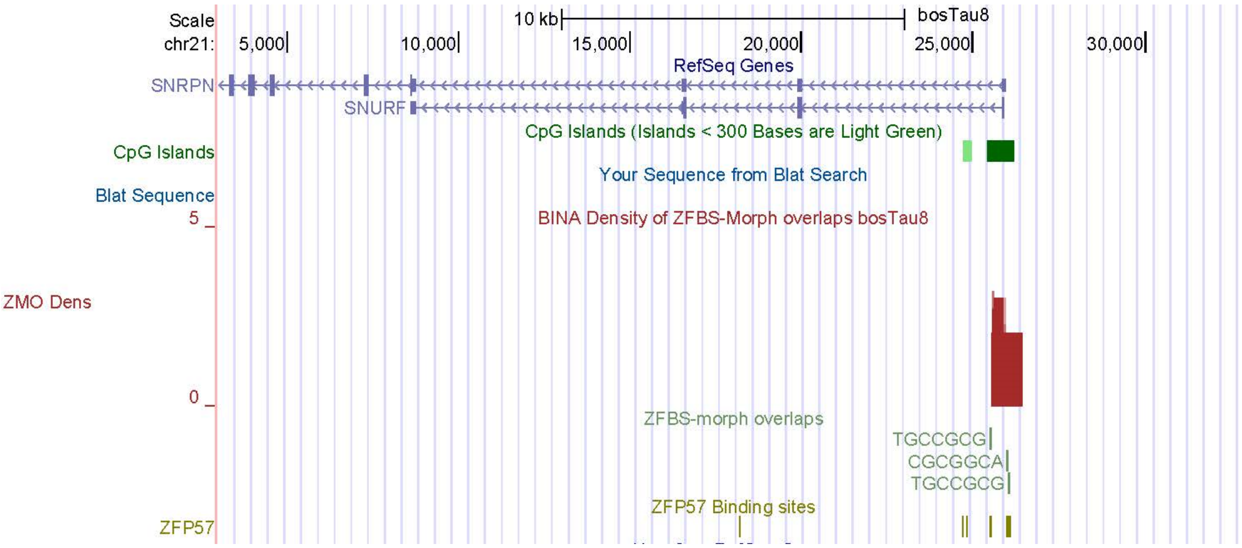

Fig. S1. A closeup view of a candidate ICR for imprinted expression of *SNRPN*, a known imprinted gene in cattle [1].

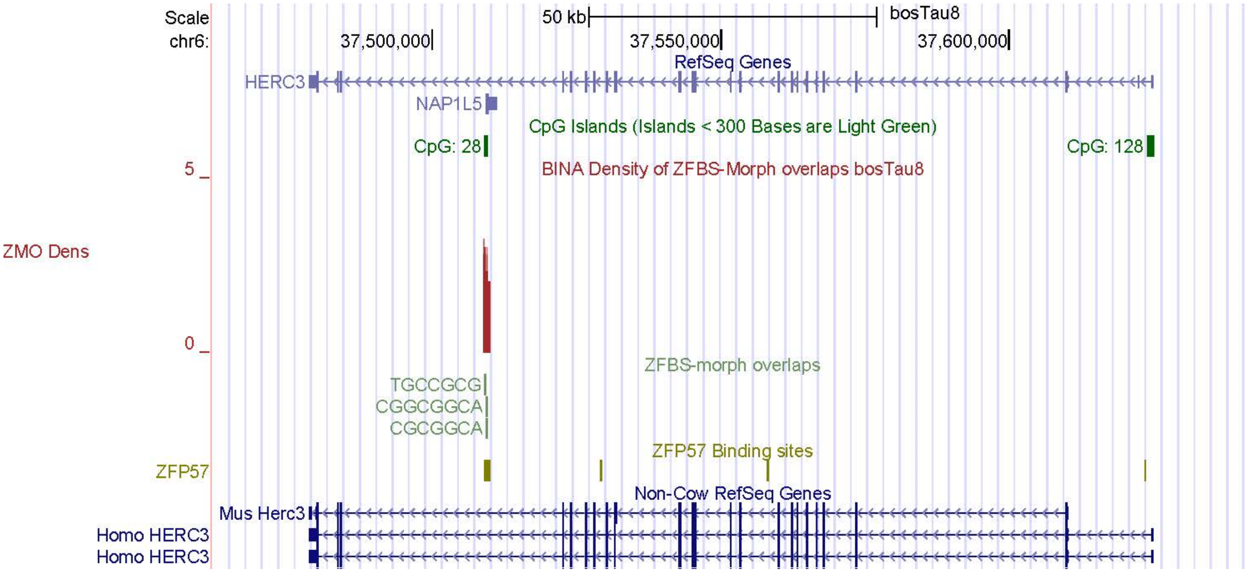

Fig. S2. A closeup view of a candidate ICR for imprinted expression of *NAP1L5*, a known imprinted gene in cattle [2].

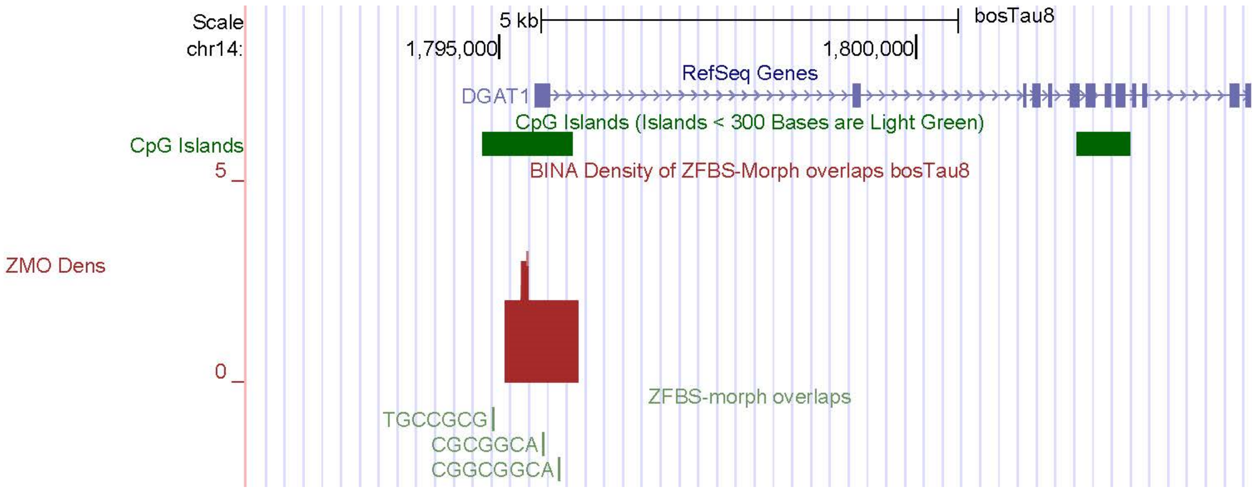

Fig. S3. A closeup view of a candidate ICR for imprinted expression of *DGAT1*. In cattle, this gene is expressed from the maternal allele [3].

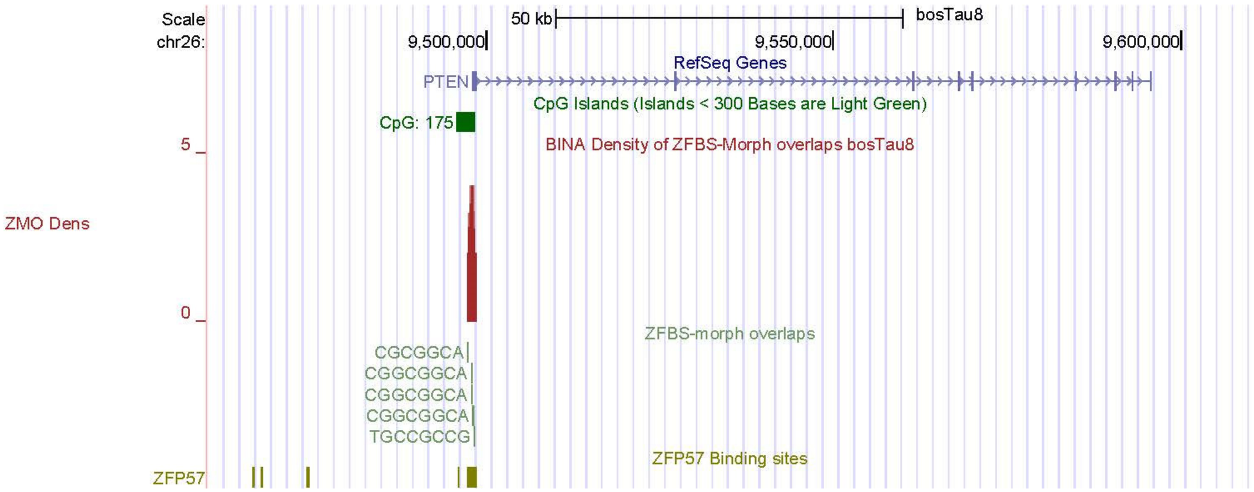

Fig. S4. A closeup view of a candidate ICR for imprinted expression of *PTEN*. Our strategy predicts that this ICR regulates the expression of a potential imprinted gene (*PTEN*) in *Bos taurus*.

References

1. Lucifero D, Suzuki J, Bordignon V, Martel J, Vigneault C, Therrien J, Filion F, Smith LC, Trasler JM: **Bovine SNRPN methylation imprint in oocytes and day 17 in vitro-produced and somatic cell nuclear transfer embryos**. *Biol Reprod* 2006, **75**(4):531-538.
2. Zaitoun I, Khatib H: **Assessment of genomic imprinting of SLC38A4, NNAT, NAP1L5, and H19 in cattle**. *BMC genetics* 2006, **7**:49.
3. Tian XC: **Genomic imprinting in farm animals**. *Annu Rev Anim Biosci* 2014, **2**:23-40.
